# *Vibrio cholerae* biofilm scaffolding protein RbmA shows an intrinsic, phosphate‐dependent autoproteolysis activity

**DOI:** 10.1002/iub.2439

**Published:** 2020-12-28

**Authors:** Manuel Maestre‐Reyna, Wei‐Cheng Huang, Wen‐Jin Wu, Praveen K. Singh, Raimo Hartmann, Po‐Hsun Wang, Cheng‐Chung Lee, Takaaki Hikima, Masaki Yamamoto, Yoshitaka Bessho, Knut Drescher, Ming‐Daw Tsai, Andrew H.‐J. Wang

**Affiliations:** ^1^ Institute of Biological Chemistry Academia Sinica Taipei Taiwan; ^2^ RIKEN SPring‐8 Center Sayo Japan; ^3^ Max Planck Institute for Terrestrial Microbiology Marburg Germany; ^4^ Department of Physics Philipps University Marburg Marburg Germany

**Keywords:** autoproteolysis, biofilm, phosphate, *Vibrio cholerae*

## Abstract

*Vibrio cholerae* is the causative agent of the diarrheal disease cholera, for which biofilm communities are considered to be environmental reservoirs. In endemic regions, and after algal blooms, which may result from phosphate enrichment following agricultural runoff, the bacterium is released from biofilms resulting in seasonal disease outbreaks. However, the molecular mechanism by which *V. cholerae* senses its environment and switches lifestyles from the biofilm‐bound state to the planktonic state is largely unknown. Here, we report that the major biofilm scaffolding protein RbmA undergoes autocatalytic proteolysis via a phosphate‐dependent induced proximity activation mechanism. Furthermore, we show that RbmA mutants that are defective in autoproteolysis cause *V. cholerae* biofilms to grow larger and mechanically stronger, correlating well with the observation that RbmA stability directly affects microbial community homeostasis and rheological properties. In conclusion, our biophysical study characterizes a novel phosphate‐dependent breakdown pathway of RbmA, while microbiological data suggest a new, sensory role of this biofilm scaffolding element.

**Abbreviations**
A.U.: Arbitrary Units
AUC: Analytical UltraCentrifugation
D‐loop: Disordered Loop conformation
Dmax: Maximum pair distance
DTT: Dithiothreitol
ESI: Electrospray Ionization
FnIII: Fibronectin III
HapA: Vibrio cholerae Hemagglutinin/protease A
HCl: Hydrochloric acid
[HMW0]: High molecular weight fragment concentration at time 0
k: autoproteolytic kinetic constant
KPi: potassium phosphate buffered to pH 8 as a mix of monopotassium and dipotassium phosphate
[LMW0]: Low molecular weight fragment concentration at time 0
[LMWt]: Low molecular weight fragment concentration at time t
MALS: Multiangle Light Scattering
MS: Mass Spectrometry
O‐loop: Ordered Loop conformation
OD280: Optical Density at 280 nanometers
PAGE: PolyAcrylamide Gel Electrophoresis
PCR: Polymerase Chain Reaction
R2: correlation coefficient
RbmA: Rugosity and biofilm structure modulator A
RbmC: Rugosity and biofilm structure modulator C
Rg: Radius of gyration
RPM: Revolutions Per Minute
SAXS: Small Angle X‐ray Scattering
SDS: Sodium Dodecyl Sulphate
SEC: Size Exclusion Chromatography
t: time
TOF: Time of flight
TR‐SAXS: Time‐Resolved SAXS
Tris: Tris(hydroxymethyl)aminomethane
WT: Wild Type

## 1 INTRODUCTION

The bacterium *Vibrio cholerae* is the causative agent of cholera, a seasonal pandemic affecting ca. 1.4–4.3 million people annually worldwide.^1^ During inter‐epidemic periods and in estuarine and brackish waters, *V. cholerae* reservoirs take the form of either free‐swimming bacteria, or, most importantly, sessile biofilms associated with different surfaces.^2^ RbmA along with RbmC and Bap1 are three matrix proteins that are key to biofilm ultrastructure.^3^, ^4^, ^5^, ^6^ Among these, RbmA surrounds cells within the biofilm, providing enhanced mechanical strength via glycan binding.^7^, ^8^, ^9^ RbmA also maintains the overall architecture of the biofilm, controlling bacterial orientation within the matrix.^4^, ^10^ Finally, RbmA proteolytic modification into RbmA* by the protease HapA is involved in biofilm maturation, by recruiting by‐stander cells into the biofilm.^11^ Recent studies have suggested that in spite of RbmA's preferred oligomeric state being dimeric, monomer‐dimer transitions regulated via a molecular switch^12^ underlay the wide array of RbmA function. Furthermore, Fong et al. noted that RbmA may undergo spontaneous degradation; however, the mechanism, triggering factor, and relevance of such a process remain unclear.^12^

While the *V. cholerae* biofilm initiation and maturation are well studied,^13^ the final stage of biofilm development, the dispersal of cells from biofilms, remains elusive. Recent observations have shown that nutrient starvation and quorum sensing jointly control *V. cholerae* biofilm dispersal,^14^ and that increased motility and the capacity to effectively swim through highly viscous media are important factors in dispersal.^15^ However, the molecular mechanisms that cause dispersal are unclear. The dispersal stage is particularly relevant, as *V. cholerae* cells released from biofilms are highly infective,^16^ and responsible for severe disease outbreaks. Interestingly, cholera outbreaks can be correlated to water eutrophication,^17^, ^18^, ^19^, ^20^, ^21^ in which normally limiting nutrients, such as inorganic phosphate, become abundant due to agricultural runoff. For example, seasonal river nutrient discharge in endemic regions has been linked to cholera epidemics.^20^, ^22^, ^23^

Here, we report that in the presence of phosphate and magnesium ions, RbmA oligomerizes in an orderly fashion which leads to autoproteolysis, in a manner reminiscent of other induced proximity activated autocatalytic processes.^24^, ^25^, ^26^ Furthermore, by constructing *V. cholerae* strains with mutations in key residues for autoproteolysis in RbmA, we observed enhanced biofilm growth capacity. In conclusion, our data suggest that phosphate sensing via RbmA autproteolysis, and the subsequent matrix weakening, may facilitate *V. cholerae* release from the biofilm. Therefore, RbmA is not only fundamental for biofilm structural integrity and central for biofilm ultrastructure, but also an active participant in dynamically shaping the bacterial community in response to outside stimuli.

## 2 EXPERIMENTAL PROCEDURES

### 2.1 Generation of RbmA mutants, production, and purification

RbmA mutants were generated from a previously existing, codon‐usage optimized artificial gene, which had been cloned into the pET28a vector.^9^ Primers for all mutants can be consulted on Table S2. RbmA* production and purification procedure was the same as previously published.^11^ The FnIII B‐domain isolate was generated by PCR based on the same full‐length construct as above, followed by re‐cloning into the pET28a vector. Protein expression and purification followed previous guidelines.^9^

### 2.2 RbmA autoproteolysis experiments

RbmA and mutants in 20 mM Tris/HCl pH 8, 100 mM NaCl were mixed with different amounts of a 0.5 M potassium phosphate solution to obtain final phosphate concentrations ranging from 1 μM to 100 mM. Divalent cations, when added, were diluted from a 100 mM stock to a final concentration of 2.5 mM. All mixtures were performed in such a way that final RbmA concentration was 5 mg/ml. All sample components were freshly sterile filtered before mixing, and all containers had been autoclaved at most 8 hr prior to starting the reaction. The reaction was started by exposing the protein mix to 37°C, and 200 rpm shaking. The 10 μg samples were periodically extracted from the reaction mix, and combined with SDS‐loading buffer +0.2% β‐mercaptoethanol under sterile conditions. These were then heated at 95°C for 5 min, and stored at 4°C until the experiment's end. Once all samples had been gathered, SDS‐PAGEs were run by loading 5 μg of each time‐dependent sample, along with 5 μL of SeeBlue® Plus2 Protein Standard (Thermo‐Fischer). Reaction rates were calculated by performing autoproteolysis experiments as described above at fixed phosphate and magnesium concentrations (10 mM potassium phosphate, 2.5 mM MgCl_2_). Relative band intensities were obtained via gel‐densitometry, using the ImageJ^27^ software, and normalizing the values to the total lane intensity of each band. Obtained values were then fitted in Qtiplot to an autocatalytic model (Equation 1).

(1) $$\left[{\mathrm{LMW}}_{t}\right]=\frac{\left[{\mathrm{LMW}}_{0}\right]+\left[{\mathrm{HMW}}_{0}\right]}{1+\frac{\left[{\mathrm{HMW}}_{0}\right]}{\left[{\mathrm{LMW}}_{0}\right]}e^{-\left(\left[{\mathrm{HMW}}_{0}\right]+\left[{\mathrm{LMW}}_{0}\right]\right)\mathrm{kt}}}.$$

### 2.3 Edman degradation

Samples for Edman degradation were prepared analogously to mass spectrometry samples. These were then sent to Mission Biotech, which performed the degradation of 300 pmoles of the C‐terminal RbmA fragment mixture via 12 cycles on an Applied Biosystems Procise Sequencer.

### 2.4 Mass spectrometry

Mass spectrometric data from RbmA autoproteolysis products were obtained by incubating RbmA under optimal conditions for 3 days at 37°C. Next, RbmA fragments were purified via size exclusion chromatography (pre‐packed HK16/60 Superdex 200 column, GE life sciences), and buffer exchanged to 20 mM Tris/HCl pH 8. Samples were then concentrated to 1 mg/ml and subjected to ESI‐TOF/MS on a Waters Synapt G2 HDMS mass spectrometer.

### 2.5 Analytical ultracentrifugation

Samples for analytical ultracentrifugation (AUC) were prepared as for autoproteolysis experiments, and were not sterile filtered or centrifuged prior to the experiments, in order to avoid elimination of low molecular weight aggregates resulting from potassium phosphate‐mediated filamentation. Samples were studied via sedimentation velocity experiments in a Beckman Coulter ProteomLab XL‐I centrifuge. Because of the high optical densities at 280 nm (OD_280_ ~ 7, for RbmA concentrations of 5 mg/ml), experiments were monitored in parallel via Absorbance at 250 nm, and Rayleigh Interference. Ultracentrifugation experiments took place at 40,000 RPM.

### 2.6 Size exclusion chromatography coupled multiangle light scattering

Size exclusion chromatography coupled multiangle light scattering (SEC**‐**MALS) measurements were carried out with a miniDAWN TREOS detector (Wyatt Technology Corporation) coupled to an Agilent 1,260 Infinity HPLC. The 240 ~ 360 μg protein samples were injected into a size exclusion chromatography column (ENrich SEC 70, Bio‐Rad) and continuously run at a flow rate of 0.5 ml/min in the buffer containing 20 mM Tris (pH 8.0), 200 mM NaCl, 2 mM DTT, and 0.02% NaN_3_. The molecular weights were determined by multi‐angle laser light scattering using an in‐line miniDAWN TREOS detector and an Optilab T‐rEX differential refractive index detector (Wyatt Technology Corporation). Bovine serum albumin (Sigma, A1900) was used for system calibration and the data were analyzed using ASTRA 6 software (Wyatt Technology Corporation) with the dn/dc value set to 0.185 ml/g.

### 2.7 RbmA protein crystallization, structure solution, and refinement

RbmA crystallization took place under previously known conditions^9^ (PDBID 4BE6), modified slightly to incorporate 10 mM magnesium sulfate. Crystals grew within 2 days, with data collected at the BL15A1 beamline, National Synchrotron Radiation Center, Hsinchu, Taiwan. Data was processed with XDS,^28^ solved via molecular replacement using two water‐stripped B‐chains from the 4BE6 structural model as search ensembles. Refinement was performed by a combination of manual evaluation via Coot,^29^ and automatic, least squares minimization via Refmac5.^30^ Initial TLS parameters were obtained via the TLS Motion Determination server,^31^ and subsequently refined both via Refmac5, and by hand. Processing and refinement data can be consulted on Table S3. The atomic coordinates for RbmA in complex with magnesium ion has been deposited in Protein Data Bank with access number 5G50.

### 2.8 Small‐angle X‐ray scattering data collection and analysis

SAXS measurements were carried out using the RIKEN SAXS beamline at BL45XU^32^ at SPring‐8 synchrotron radiation facility (Hyogo, Japan). The beam wavelength was set to 1 Å. The DECTRIS PILATUS 3X 2 M detector was positioned at a distance of 2.5 m from the sample, with the direct beam off‐centered.

The time‐resolved SAXS experiments were performed by coupling a stopped‐flow instrument (Unisoku Co. Ltd., Japan) immediately prior to the SAXS measurement. RbmA samples at 10 mg/ml in 20 mM Tris/HCl pH 8, 100 mM NaCl, and buffers including or excluding double concentration of phosphate and magnesium were loaded into a stopped‐flow instrument, which, after triggering the mixing resulted in 5 mg/ml RbmA in 20 mM Tris/HCl pH 8, 100 mM NaCl, with or without 10 mM potassium phosphate and 2.5 mM MgCl_2_. The scattering data were collected with an exposure time of 0.2 s every 36 s in a period of 3 hr.

The size‐exclusion‐chromatography in line with SAXS experiments (SEC‐SAXS) was performed by connecting an analytical Superdex 200 increase 3.2/300 mm column immediately prior to the SAXS sample capillary. Samples were passed at a flow rate of 0.075 ml/min, and the scattering data were collected with an exposure time of 1 s for every 3 s.

Data processing, analysis, and modeling steps were carried out using the ATSAS suite.^33^ The radius of gyration (*R*_*g*_) was derived via AutoRg^34^ using the Guinier approximation.^35^ The indirect Fourier transform method was utilized to calculate the pair‐distance distribution function (*p[r]*), *R*_*g*_, and *D*_max_ using the DATGNOM4 software.^36^

## 3 MODELING SAXS PROFILES OF THE DIFFERENT POSSIBLE RBMA FILAMENTOUS FORMS

The magnesium bound crystal structure presented in this manuscript (PDB ID 5G50) was propagated either along its tight or wide groove faces via usage of the translate command in the PyMOL software,^37^ followed by calculation of a SAXS profile via the FoXS program.^38^ *R*_*g*_ and *D*_max_ parameters were then generated as above for the experimental data.

### 3.1 Thermofluor‐assay experiments

In order to perform protein stability assays, we followed previously described protocols closely.^39^ Briefly, RbmA wild type (WT) and mutant stocks were diluted to 1 mg/ml and combined with 100 fold diluted sypro‐orange dye. Samples were then exposed to a 1°C/min temperature increase, with fluorescence intensity monitored as a function of time/temperature.

## 4 STRAIN CONSTRUCTION

All *V. cholerae* strains used for biofilm experiments were derivatives of the WT C6706 (quorum sensing capable variant),^40^ which is an O1 El Tor biotype. The *rbmA* ^*E160A*^ and *rbmA* ^*W222G*^ alleles were generated using a sewing PCR method. Fragments of approximately 1 kb upstream (including 5′ *rbmA*) and 1 kb downstream (including 3′ *rbmA*) of *rbmA* were polymerase amplified. Different pairs of oligonucleotides were used for generating point mutations (given in Table S4). Sewing PCR was used to join the upstream and downstream PCR fragments. Final PCR products were digested with NotI and NheI restriction enzymes and ligated into an appropriately digested plasmid pNUT144, which was described previously,^41^ based on the pKAS32 vector^42^). The resulting ligation mixtures were used to transform the WT *E. coli* S17 strain. Positive transformants were first selected by colony PCR, and later confirmed by sequencing. Plasmids containing *rbmA* ^*E160A*^ and *rbmA* ^*E160A*^ mutants were named pNUT1007 and pNUT1009, respectively.

Mating of both the plasmids (pNUT1007 and pNUT1009) into *V. cholerae* strain C6706 was performed as described earlier.^41^ Sequencing was used to confirm the allelic replacement of WT *rbmA* to respective *rbmA* mutations. Final strains were named as KDV599 (*rbmA* ^*W222G*^) and KDV703 (*rbmA* ^*E160A*^). P_*tac*_‐*sfgfp* was integrated at the *lacZ* locus in both *V. cholerae* strains using the plasmid pNUT480, which is based on pNUT129,^41^ but with the *mKO* gene replaced by the *sfgfp* gene.

### 4.1 Biofilm growth experiments

Biofilms of the three strains (strain KDV428 with WT RbmA; KDV599 with RbmA^W222G^; KDV703 with RbmA^E160A^) were grown in M9 minimal media (40 mM Na_2_HPO_4_, 20 mM KH_2_PO_4_, 9 mM NaCl, 11 mM [NH_4_]_2_SO_4_, 0.1 mM CaCl_2_, 1 mM MgSO_4_, 0.06 mM FeCl_3_), pH 7.2, supplemented with 30 mM glucose. Biofilms were grown at room temperature (24 ± 1°C) on glass surfaces in microfluidic flow chambers, in which a constant flow of nutrients across the biofilms is provided.^4^ The flow chamber cross section was 500 x 100 μm^2^, and the flow rate of M9 medium through these channels was 0.1 μm/min. Images were acquired on a Nikon Ti‐E confocal microscope, and image analysis and biofilm volume calculations were performed using the BiofilmQ image analysis software.^43^

## 5 RESULTS AND DISCUSSION

### 5.1 RbmA autoproteolytically degrades in the presence of magnesium phosphate

**FIGURE 1:**
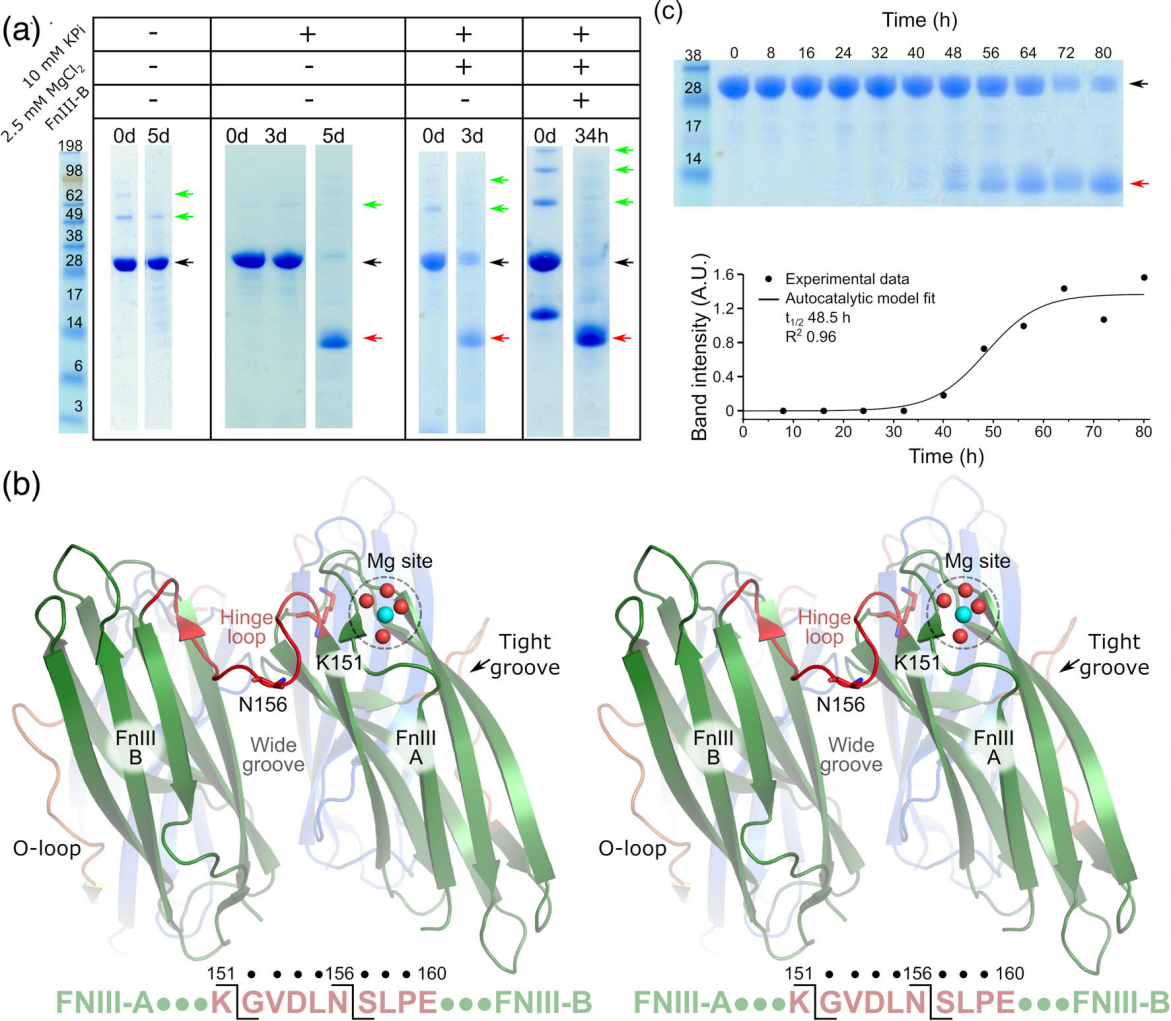
RbmA phosphate‐mediated autoproteolysis. (a) SDS‐PAGE showing RbmA incubated at 37°C with neither potassium phosphate (KPi) nor MgCl2, with 10 mM KPi, with 10 mM KPi and 2.5 mM MgCl2 (i.e., MgPO4), or with both salts and 1 mg/ml FnIII B‐Domain (WT + FnIII B). Lanes show different digestion times. Black arrows correspond to the intact RbmA, while red to the degradation fragments. Note that some higher order RbmA oligomers survived sample treatment, and are marked with green arrows. (b) Stereo representation of the overall RbmA topology and autoproteolysis cutting site. N‐terminal sequencing combined with mass spectrometry revealed that the two mass peaks (Figure S2A) correspond to C‐terminal fragments ranging from either K151 or N156 to the C‐terminus. Furthermore, by adding MgSO4 to our crystallization conditions, we observed a magnesium binding site on the surface of the FnIII A‐domain. The RbmA dimer is presented in green and blue, corresponding to each polypeptide chain. (c) Autocatalytic reaction kinetic of wild type RbmA in the presence of 10 mM KPi and 2.5 mM MgCl2. The graph shows individual time‐points, densitometrically measured from the gel above, and their corresponding autocatalytic model fit (solid line)

RbmA is a very stable protein, remaining intact at 37°C for at least 1 week (Figure 1a). However, we discovered that in the presence of phosphate, the protein underwent slow degradation, as demonstrated by the gradual disappearance over time of the main 28 kDa RbmA band and simultaneous appearance of a 13 kDa low molecular weight band in our SDS‐PAGEs (Figure 1a). Mass spectrometry data revealed that the low molecular weight band was composed of two products, a 12.5 and a 13 kDa fragment (Figure S1a). Edman degradation experiments indicated that these two degradation fragments both corresponded to an RbmA's C‐terminal domain fragment (the FnIII B‐domain), and that their cutting sites were located in the protein hinge region, after amino acids K151 and N156, respectively (Figure 1b). Furthermore, AUC revealed the fragment to be monomeric in nature (Figure S1b).

Furthermore, screening against different divalent cations and phosphate concentrations indicated that phosphate‐dependent degradation was accelerated by magnesium (Figures 1a and S2). In the absence of magnesium, phosphate elicited significant protein degradation within 5 days. However, addition of 2.5 mM MgCl_2_ resulted in degradation products appearing in 3 days (Figure 1a). We have also monitored the accelerated reaction every 8 hr (Figure 1c), with products appearing within 48 hr. This behavior could be observed even at low concentrations of phosphate (10 μM, Figure S2). Conversely, the protein remained stable when exposed to 2.5 mM MgCl_2_ and 1 μM phosphate (Figure S2).

Time‐dependent kinetic analysis of the degradation reaction (Figure 1c, Table S1) resulted in a sigmoidal curve which could be best fitted via an autocatalytic model^44^ (half‐life of 51.3 ± 2.9 hr). By contrast, a non‐autocatalytic model catalyzed by a putative protease contaminant would not follow an autocatalytic kinetic, but rather a pseudo‐first order kinetic, as RbmA would be in great excess over the protease. The overall reaction could not be fitted by such a model, and adopting an initial velocities approach^45^ yielded similarly poor statistics (*R* ^2^ correlation coefficients of 0.86 and 0.88). Thus, the data suggest that no external protease is acting on RbmA, but that autoproteolysis was taking place.

In an autocatalytic reaction, the product (the FnIII B‐domain) is the fully active enzyme, that is, the high‐efficiency catalyst responsible for subsequent reaction cycles. In support of such a mechanism, we observed that when a 1:1 M mix of WT RbmA and separately purified RbmA FnIII B‐domain was exposed to 10 mM phosphate and 2.5 mM MgCl_2_, the reaction became faster, with all protein molecules being processed within 34 hr (Figure 1a).

Overall, these data show that RbmA undergoes autoproteolysis via activation of its FnIII B‐domain, but does not explain how the reaction is initiated by magnesium phosphate, a topic which we analyze in the following sections.

### 5.2 RbmA oligomerizes in the presence of magnesium phosphate

**FIGURE 2:**
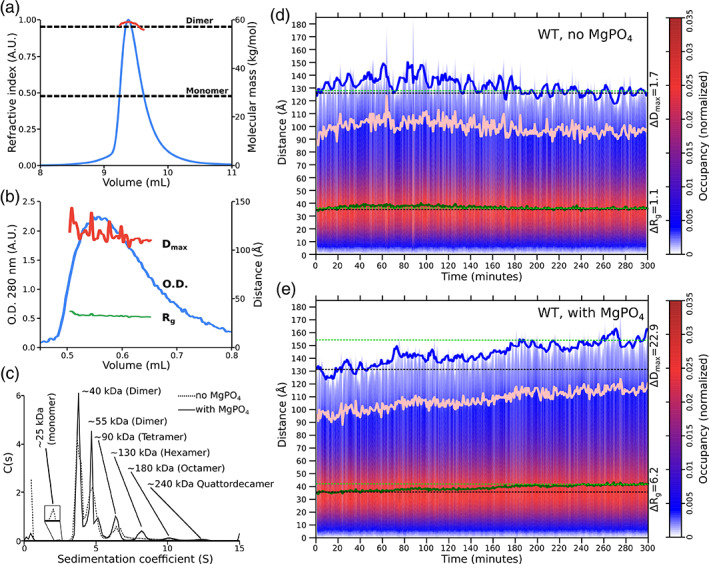
Investigating RbmA oligomerization. (a) SEC‐MALS chromatogram showing the refractive index trace (blue) and the corresponding molecular weight around the peak (red) in the absence of MgPO4. For orientation, dotted lines indicate the calculated molecular weights of the RbmA dimer and monomer. (b) SEC‐SAXS chromatogram showing the optical density at 280 nm in blue, while the peak radius of gyration (*R*_*g*_) in green, and the maximum distance (*D*_max_) in red in the absence of MgPO4. (c) AUC traces for RbmA with (solid line) and without (dotted line) MgPO4. Assigned oligomeric state for each peak is described near each of them. In order to highlight the very small monomeric peak found in the absence of MgPO4, its intensity was increased 15‐fold in a small panel near it. (d) Time‐dependent SAXS pair distance distribution heat map for RbmA in the absence of MgPO4. Here, the probability for a given pair distance is given by the color (red for high probability, blue for low probability) as indicated by the nearby scale bar. Five point running averages for the *R*_*g*_ (defined as the distance with the highest probability) is shown as a green trace, while for *D*_max_ (defined as the highest distance value) in blue. To mark the overall spread of the data, initial (black) and final (green) five point averages for *R*_*g*_ and *D*_max_, are shown as dotted lines. Finally, because, due to its inherent low probability, *D*_max_ values tend to fluctuate strongly, the 90% distance is shown as a solid pink line to highlight the significance of *D*_max_ change. Differences between initial and final values of *R*_*g*_ and *D*_max_, Δ*R*g and Δ*D*_max_, respectively, are shown nearby. (e) Time‐dependent SAXS pair distance distribution heat map for RbmA in the presence of MgPO4. Plot is shown as in (d)

By definition, in an autocatalytic reaction (A + B ➔ 2B) individual molecules do not self‐catalyze their reaction, but some molecules act as a catalyst *B* on others, which behave as substrate *A*.^46^ These are then converted themselves into a catalyst *B* as a result of the reaction. Accordingly, initiation of an autocatalytic reaction requires either a small amount of product/catalyst *B* to be present in the mix, which agrees with RbmA's accelerated proteolysis in the presence of the FnIII‐domain (Figure 1a), or for a triggering event to increase the probability of a spontaneous conversion of inactive *A* into catalytic *B*. One such triggering factor in many autoproteolytic systems is transient oligomerization during the initial stages of proteolysis, a mechanism called induced proximity activation.^24^ Thus, we decided to test if RbmA presented MgPO_4_‐dependent oligomerization. In previous work, and in the absence of MgPO_4_, RbmA has been repeatedly shown to exist predominantly as a dimer, both in solution, and *in crystallo*.^8^, ^9^, ^12^ We could confirm this behavior via SEC‐MALS experiments, as a molecular weight of 57.6 kDa was determined for the single elution peak (Figure 2a), which is almost precisely double the expected molecular weight of the RbmA monomer (28.6 kDa). A similar result could be obtained via SEC coupled small angle X‐ray scattering (SEC‐SAXS), which showed a single peak with a radius of gyration (*R*_*g*_) of 32.9 ± 1.4 Å (Figure 2b). As previously published,^8^ the *R*_*g*_ of RbmA is much larger than expected from calculations based on its crystal structure (20 Å for the crystallographic monomer, and 23 Å for the dimer), a feature which had been assigned to RbmA opening along the wide groove in solution (Figure 1b), adopting an extended conformation.

More sensitive AUC analysis in the absence of MgPO_4_, however, highlighted the presence of a very small population of monomeric RbmA, as well as more abundant higher molecular weight species (Figure 2c), which were assigned as one tetrameric and two alternative dimeric species, the latter possibly due to geometric effects on sedimentation speed related to the open versus closed conformations available to RbmA dimers.^8^, ^9^ Indeed, SDS‐PAGE analysis (Figure 1a) shows such higher order supramolecular elements survive the sample preparation process, resulting in high molecular weight bands on the gel lanes. Importantly, when phosphate and magnesium was added to the RbmA solution, even higher molecular weight species appeared in the AUC profile (Figure 2c), indicating that phosphate and magnesium do elicit oligomerization and/or aggregation of RbmA. Therefore, our data suggest that RbmA may initiate its autoproteolytic behavior via an induced proximity activation mechanism.

### 5.3 RbmA oligomers are well‐ordered and filamentous in nature

A crucial feature of induced proximity activation processes is that, rather than being brought about by amorphous aggregation, they usually involve well‐ordered oligomerization.^24^, ^25^, ^26^ In order to determine if RbmA oligomers grew in an ordered fashion, we performed time resolved SAXS (TR‐SAXS) experiments, which provide time‐dependent information of particle shape in solution via analysis of the pair distance distribution function^47^ (Figures S3 and S4). Here, two key parameters were calculated, namely the radius of gyration (*R*_*g*_) which corresponds to the average intramolecular distance, and is proportional to overall particle size, and the maximum intramolecular distance *D*_max_, which directly correlates to how elongated a particle is.^47^ The expectation is that, during symmetric, amorphous growth, particles will become larger and more spheric, as they grow equally in all directions, and therefore *D*_max_ and *R*_*g*_ tend to grow at similar rates (Figure S3a). Conversely, during asymmetric filamentous growth, the average intramolecular distance will still be mostly determined by the width of the filament subunits, while elongation results in dramatically increased maximum distance. Accordingly, the hallmark of filamentous growth is a small change in *R*_*g*_, while a large increase in *D*_max_ (Figure S3b).

Here, upon addition of MgPO_4_ to RbmA, a moderate increase in *R*_*g*_ could be detected (Δ*R*_*g*_ = 6.2 Å); however, at the same time, *D*_max_ grew dramatically within minutes to hours (Δ*D*_max_ = 22.9 Å, Figure 2d,e) which supports asymmetric elongation of the RbmA particles. Particle elongation is strong evidence for ordered oligomeric structures, as asymmetric growth at the molecular level can only happen if specific RbmA faces are interacting with each other in a regular fashion. Furthermore, the observed time and MgPO_4_‐dependent changes in RbmA WT SAXS parameters are consistent with the formation of filamentous hexamers, as a hexameric model based on the crystallographic structures resulted in a Δ*R*_g_ of 9 Å and Δ*D*_max_ of 22 Å versus the experimental dimer.

Next, we hypothesized that, if MgPO_4_ triggered ordered oligomerization in RbmA, then the protein should present specific binding sites for it. Indeed, co‐crystallization with MgSO_4_, which acted as a non‐catalytic analogue of MgPO_4_, allowed us to identify a single, loosely coordinated magnesium atom bound to the FnIII A‐domain via the carbonyl backbone of residue Q50, the side‐chain of residue Q48, and four water molecules (Figure 1b). Interestingly, the magnesium binding site was part of a crystal lattice contact between the FnIII A‐ and B‐domains of different asymmetric unit molecules (Figure S5), indicating that the presence of magnesium does promote intermolecular interactions.

In summary, SAXS and crystallographic data strongly support the idea that MgPO_4_ promotes RbmA oligomerization, and with it, initiates autocatalytic proteolysis via an induced proximity mechanism.

### 5.4 Searching for critical residues in RbmA autocatalysis

**FIGURE 3:**
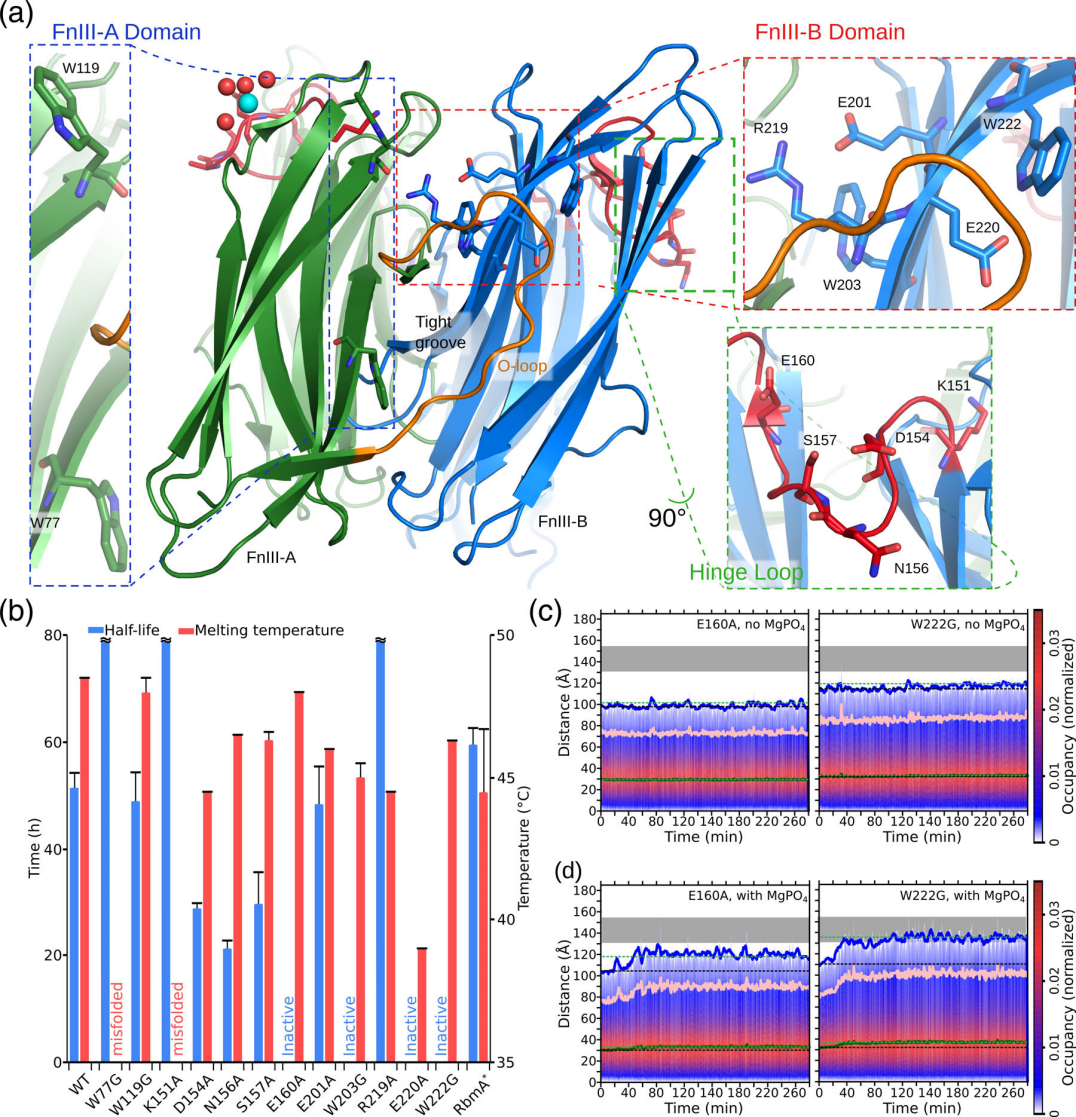
Mutational analysis of RbmA autoproteolysis and oligomerization. (a) Location of the planned mutants on the RbmA structure. The tight groove of the RbmA dimer is shown with one monomer colored green and the other blue. Regions targeted for mutation are zoomed in and individual amino‐acids shown as stick models. For a stereo view of this figure, please consult Figure S9. (b) Bar diagram presenting the autoproteolysis half‐lives (blue bars) and thermal stability (red bars) for the wild type (WT) as well as each generated RbmA mutant. Standard deviations are shown as whiskers on top of each bar. Very long half‐lives that exceeded the experiments length are shown as truncated blue bars (W77G, K1151A, and R219A). When no reaction was observed, “inactive” is written instead of providing a half‐life. Furthermore, when no melting temperature could be calculated, “misfolded” was written instead of providing a temperature. (c) Time‐dependent SAXS pair distance distribution heat map for RbmA mutants in the absence of MgPO4. Here, the probability for a given pair distance is given by the color (red for high probability, blue for low probability) as indicated by the nearby scale bar. Five point running averages for the *R*_*g*_ are shown as a green trace, *D*_max_ in blue, and 90% *D*_max_ in pink. To mark the overall spread of the data, initial (black) and final (green) five point averages for *R*_*g*_ and *D*_max_, are shown as dotted lines. For comparison with the wild‐type, the WT Δ*D*_max_, in the presence of MgPO4 is shown on the plots as a grey square. (d) Time‐dependent SAXS pair distance distribution heat map for RbmA mutants in the presence of MgPO4. Plots are presented as in (c)

In order to find which elements of RbmA are critical during its MgPO_4_ induced proximity autoproteolytic behavior, we generated a number of mutants (Figure 3). One obvious region to target was the hinge loop, as it is the subject of cleavage (Figure 1b). Accordingly, we generated point mutations for all polar residues in the region (Figure 3). Additionally, from all the possible multimer orientations that we modeled, those where RbmA dimers interacted via their tight groove faces fit the TR‐SAXS data best, leading us to target it for mutagenesis (Figure 3a). One interesting feature of the tight groove face is that a long FnIII A‐domain loop may either hug the FnIII B‐domain across the domain‐domain interface (O‐loop conformation), or transition into a disordered state^9^ (D‐loop conformation). Although, in all published crystal structures, the O‐loop conformation is adopted within an individual RbmA dimer, we hypothesized that, if RbmA oligomers interacted via the tight groove face, the O‐loop conformation could act as a cross‐dimer interaction. Thus, we targeted those residues which were responsible for stabilizing the O‐loop conformation (E201, R219, E220, and W222, Figure 3a). In addition to W222, the final set of mutagenesis targets were tryptophans 77, 119, and 203, which are all conspicuously located on the solvent exposed surface of the tight groove, an arrangement which has been demonstrated to promote attachment to hydrophobic surfaces and oligomerization in other glycan binding proteins.^48^, ^49^

Proteolytic assays performed with these mutants (Figures 3b and S6 and Table S1) narrowed down our candidates to E160A, in the hinge loop, and W203G, E220A, and W222G in the FnIII B‐domain, as they were all incapable of it. From these, E220A was discarded because its structural integrity was compromised, as demonstrated via thermofluorassay heat denaturation experiments, where its melting temperature was 10°C lower than the WT (Figure 3 and Table S1). We also did not further characterize W203G.

On the other hand, TR‐SAXS data from the remaining two mutants (E160A and W222G) revealed that, in the absence of MgPO_4_ they both presented a markedly more compact shape than the WT (Figures 3c vs. 2d). Furthermore, addition of MgPO_4_ (Figure 3d) resulted in both mutants suffering a conformational change within the first 40 min of reaction time, which lead them to adopt *R*_*g*_ and *D*_max_ parameters similar to the MgPO_4_‐free WT. From that point on, however, no further changes occurred in either mutant, which did not continue to grow as the WT had done (Figure 3d vs. 2e). A comparison with calculated SAXS parameters suggested that, in the absence MgPO_4_, both mutations existed in a similar conformation to the closed crystallographic dimer. The comparatively rapid conformational change elicited by the addition of MgPO_4_ counteracted this effect of the mutations, bringing both closer to the MgPO_4_‐free WT dimer, in which opening of the wide groove caused it to have a more elongated shape than found *in crystallo*. This may be explained by the increased ionic strength of the MgPO_4_‐containing solution, as most wide groove interactions are salt bridges, which are highly sensitive to the salt concentration of the medium.^50^ However, after the opening of the wide groove, neither of the autocatalytically inactive mutants appears to have been capable of following the oligomerization process as the WT did, suggesting a link between oligomerization and autoproteolysis initiation.

**FIGURE 4:**
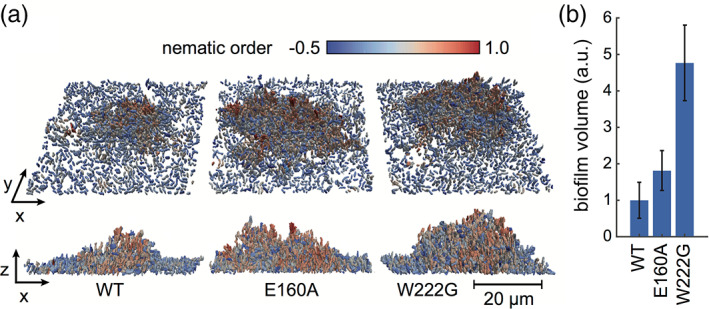
Effects of autoproteolysis‐defective RbmA mutants on biofilm growth. (a) *V. cholerae* biofilms were grown for 24 hr and then imaged in 3D using single‐cell confocal microscopy. Renderings show biofilm architecture for the WT, RbmA‐E160A, and RbmA‐W222G strains. Each cell is colored according to the local alignment with its neighbors, quantified using the nematic order parameter (blue indicates no alignment, red indicates high alignment). (b) Biofilm volumes were calculated from the 3D image data, normalized to the WT biofilm volume

In good agreement with these data *V. cholerae* strains expressing these two non‐autocatalytic mutants grew into larger biofilms that were more resistant to fluid‐shear than WT biofilms, with enhanced biofilm growth by a factor of 2 and 5, for E160A and W222G, respectively (Figure 4a,b). These experiments were conducted in M9 medium containing a high concentration of phosphate, for which the biophysical experiments have shown that it leads to autoproteolysis. The low shear‐resistance of WT biofilms compared with the E160A and W222G strains is consistent with the hypothesis that autoproteolysis influences biofilm stability. In low phosphate conditions it was not possible to grow biofilms so that direct observations of the effect of these RbmA mutations on dispersal were not possible. However, it cannot be ruled out that the increased biofilm volume growth of these RbmA mutants is the result of an increased production of VPS. Yet when these mutants were inspected for colony rugosity after 24 and 72 hr of growth (Figure S7), which is a phenotype that requires elevated VPS production, the RbmA mutants presented the same non‐rugose colony phenotype as the WT. In contrast, it has been shown that the FnIII B‐domain only partially complements RbmA negative *V. cholerae* strains,^12^ indicating that both RbmA domains are required for full strength biofilm scaffolding.

Thus, mutational analysis assigns a critical role for hinge loop residue E160 during RbmA autoproteolysis, while further confirming that the FnIII B‐Domain is key to autoproteolysis via its tight groove side, where W222 is located. Furthermore, W222 has an important role in stabilizing the O‐loop conformation via its interactions with the V94‐G95‐V96 hydrophobic patch of the O‐loop (Figure S8). Together with W222G's impaired filamentation capacity, this suggests that RbmA oligomerization relies on the D‐ to O‐loop transition to achieve induced proximity activation.

### 5.5 RbmA dimerization facilitates autoproteolysis

**FIGURE 5:**
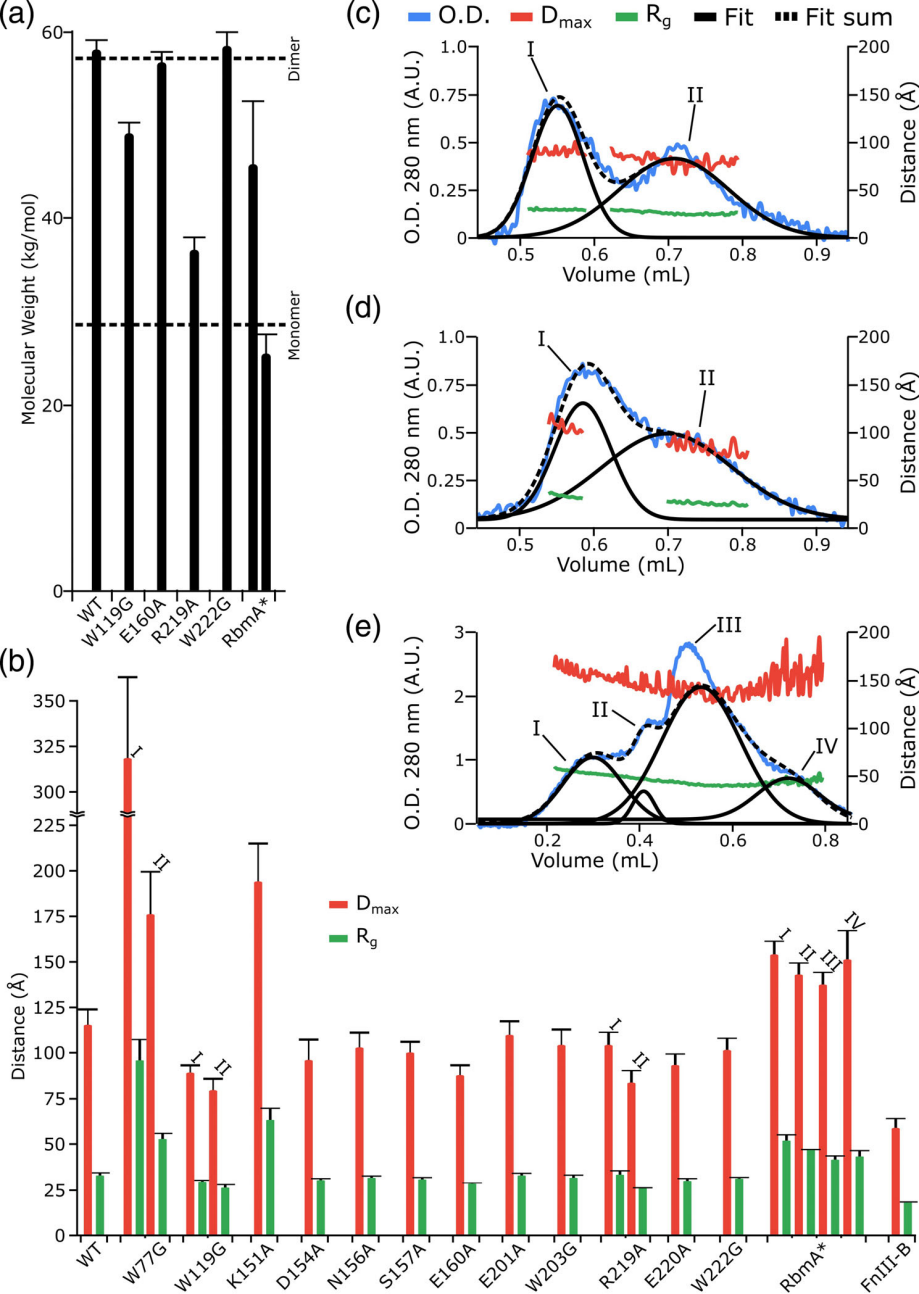
Analysis of monomer‐dimer equilibrium in RbmA mutants. (a) SEC‐MALS derived average molecular weights for RbmA wild type (WT), selected mutants and RbmA*. For comparison dotted lines indicate the expected sizes of the WT dimer and monomer. (b) SEC‐SAXS parameters for all RbmA isoforms. SEC‐SAXS derived *D*_max_ (red) and *R*_*g*_ (green) are shown in bar diagrams. When more than one peak eluted from the size exclusion column, the bars are labeled with a roman numeral in order of elution. For the extremely large W77G peak I particle, the bar has been truncated above 225 Å. (c) SEC‐SAXS profile of the W119G mutant, showing the double peak elution profile, with optical density at 280 nm (O.D.) in blue, peak *D*_max_ in red and peak *R*_*g*_ in green. Peaks are numbered with a roman numeral as in (b). In order to determine the precise position and relative size of the peaks, a multi‐Gaussian fit was performed, with individual curves shown as solid black lines, while the fit sum as a dotted line. (d) SEC‐SAXS profile of the R219A mutant, presented as in (c). (e) SEC‐SAXS profile of RbmA*, presented as in (c) and (d)

Up to this point, our studies have focused on the full length RbmA in its dimeric form, even though it had been previously shown that, in order to fulfill its biofilm scaffolding function, RbmA must exist in a dimer: monomer equilibrium.^12^ Accordingly, it is worth analyzing if monomer‐dimer transitions affect RbmA autoproteolysis. Here, SEC‐MALS experiments showed that out of all analyzed mutants, only W119A and R219A presented a significant monomeric fraction, as their average molecular weights were 49.9 and 34.3 kDa, respectively, versus 57.9 kDa for the WT (Figures 2a and 5a). This was further confirmed via SEC‐SAXS, where the WT eluted as a single peak with an *R*_*g*_ of 31.3 ± 1.4 Å and *D*_max_ of 116.6 ± 8.2 Å (Figure 2b), while W119G and R219A eluted as two peaks (peaks I and II, Figure 5b–d). Their peak I *R*_*g*_ and *D*_max_ of 29.5 ± 0.6 and 89.8 ± 4.0 Å (W119G) and 33.5 ± 1.9 and 105.9 ± 5.9 Å (R219A), overlaps well with both the experimental WT data, and with an extended dimer model (*R*_*g*_ ~ 30 Å, *D*_max_ ~ 100 Å). Nonetheless, their peak II *R*_*g*_ and *D*_max_ values of 26.2 ± 1.8 and 80.8 ± 5.6 Å (W119G), and 25.8 ± 1.5 and 83.9 ± 7.0 Å (R219A), respectively, is closer to our predicted extended RbmA monomer *R*_*g*_/*D*_max_ values of 25 Å/90 Å. Furthermore, numerical integration of peaks I and II (Figure 5c,d) allowed us to determine that the dimer: monomer molar ratio was 1:2.5 for W119G, while 1:3.5 for R219A, suggesting that R219A is more detrimental to dimerization than W119G. Interestingly, W119G, with its more dimeric population, shows autoproteolytic behavior similar to the WT (Figure 3b), while, for the more monomeric R219A, degradation is much slower (half‐life of over 80 hr, Figure 3b). To us, these data suggest that, unlike previous limited proteolysis experiments, where trypsin mediated proteolysis was more effective for monomeric RbmA,^12^ RbmA autoproteolysis necessitates the dimeric state to be initiated.

### 5.6 Effects of RbmA post‐translational maturation on autoproteolysis

In addition to being dependent on its oligomerization patterns, RbmA function is also mediated in vivo via partial proteolysis by extracellular protease HapA, resulting in the truncated RbmA*, which has important implications in the later stage of biofilm recruitment.^11^ Therefore, it is important to examine the effect of MgPO_4_ on RbmA* stability. In fact, we observed that RbmA* was also degraded in the presence of MgPO_4_ with several intermediate stages (Figures 3b and S6e). The appearance of such intermediate species is a clear indication that, although the end product is the same (the FnIII B‐Domain fragment), the autoproteolysis kinetics in RbmA and RbmA* are not. One possible explanation here is that in RbmA*, more potential autoproteolytic cleavage sites are exposed, as RbmA post‐processing by HapA into RbmA* takes place by cleavage of a 13 kDa N‐terminal peptide.^11^ This does not affect the integrity of the FnIII B‐domain, but it does cause partial unfolding of the N‐terminal FnIII A‐Domain,^12^ as confirmed by SEC‐SAXS, where, although the RbmA* is smaller than RbmA (Figure 5a), it elutes in several peaks with significantly increased *R*_*g*_ and *D*_max_ parameters (Figure 5b,e). Thus, some newly exposed polypeptide regions in RbmA* could be better targets for FnIII B‐domain mediated proteolysis than the hinge loop, giving rise to the intermediate species. Nevertheless, RbmA* is digested at a similar speed as RbmA (Figure 3b) indicating that post‐translational processing of RbmA to RbmA* does not affect MgPO_4_‐dependent degradation significantly and that, therefore, all in vivo occurring RbmA species are susceptible to it.

### 5.7 A model for RbmA proximity induced autoproteolysis

Although protein activation via slow autoproteolytic activity is common in proteases, such as *V. cholerae* HapA,^51^ it is rare in other protein types. However, several extracellular proteins, such as endolysins^52^ or complement protein C3,^53^ have been described as activating via low‐efficiency autoproteolysis. In such cases, alternative oligomerization patterns can be very important for catalysis, which also appears to be the case for RbmA, with phosphate increasing the protein's tendency for self‐assembly and oligomerization into elongated particles (Figure 2e). The most likely interaction area here appears to be the tight groove face of the RbmA dimer, as suggested by the presence of an intermolecular magnesium coordination site (Figure 1b), and by the mutation of W222, which results in a structurally intact, but catalytically inert mutant (Figure 3b). As this surface is mostly positively charged,^9^ the role of phosphate may be neutralization of positive charges to allow for intermolecular interactions to take place, although, at this point, an acid–base catalysis role for phosphate cannot be discarded.

Sustained growth of the filament is crucial for initiating RbmA autocatalysis, a process which takes minutes to hours (Figure 2e), and which ultimately leads to the production of the fully active particle, that is, the FnIII B‐domain (Figure 1) via intermolecular attack of the hinge loop. From this point on, the FnIII B‐domain becomes the main proteolytic agent, and subsequent reaction cycles are mostly mediated by it.

## 6 SUMMARY AND CONCLUSION

In the present work, we have shown that the biofilm scaffolding protein RbmA is capable of autoproteolytic self‐degradation, and we have posited that the most likely mechanism by which this happens is proximity induced activation. Self‐amplifying processes like the one presented here have been speculated to be involved in extracellular matrix unraveling,^54^, ^55^ which is an idea that is supported by non‐proteolytic RbmA mutants increasing the durability of their biofilms (Figure 4). In summary, considering that phosphate is one of the limiting growth factors for microorganisms in aquatic ecosystems,^56^ and that concentrations of phosphorous above 1 μM lead to eutrophication,^57^ which in turn has been correlated to cholera outbreaks,^58^ our data suggest a way by which degradation of RbmA may be linked to phosphate presence in aquifers. On the other hand, the relatively high abundance of magnesium in both sea water^59^ (~50 mM) and fresh water (0.1 to 15 mM)^60^, ^61^ suggests that, although important during RbmA autoproteolysis, magnesium availability may not be a determining environmental factor for triggering autoproteolysis in the estuarine and brackish environments where VPS‐dependent biofilms are most wide‐spread.^62^

We therefore propose that RbmA's MgPO_4_‐dependent autoproteolysis may function as a phosphorus sensor in *V. cholerae* biofilms, that is, a priming mechanism during micro‐colony formation that may facilitate colony dispersal.

## CONFLICT OF INTEREST

The authors declare that they have no competing interests as defined by IUBMB Life, or other interests that might be perceived to influence the results and/or discussion reported in this article.

## Supporting information


**Data S1**: Supplementary Information
