# Supplementary material for: Vibrio cholerae biofilm scaffolding protein RbmA shows an intrinsic, phosphate‐dependent autoproteolysis activity
Source: IUBMB Life. 2020 Dec 28;73(2):418–31. doi: 10.1002/iub.2439 (PMC7898620; doi:10.1002/iub.2439)
Supplement: Supplementary file 1 — Data S1: Supplementary Information [file IUB-73-418-s001.docx]

**Supplementary Materials for**

***Vibrio cholerae* biofilm scaffolding protein RbmA shows an intrinsic, phosphate dependent autoproteolysis activity.**

Manuel Maestre-Reyna^1^, Wei-Cheng Huang^1,2^, Wen-Jin Wu^1^, Praveen K. Singh^3^, Raimo Hartmann^3^, Cheng-Chung Lee^1^, Po-Hsun Wang, Takaaki Hikima^2^, Masaki Yamamoto^2^, Yoshitaka Bessho^1,2^, Knut Drescher^3,4^, Ming-Daw Tsai^1^, and Andrew H.-J. Wang^1^*

**
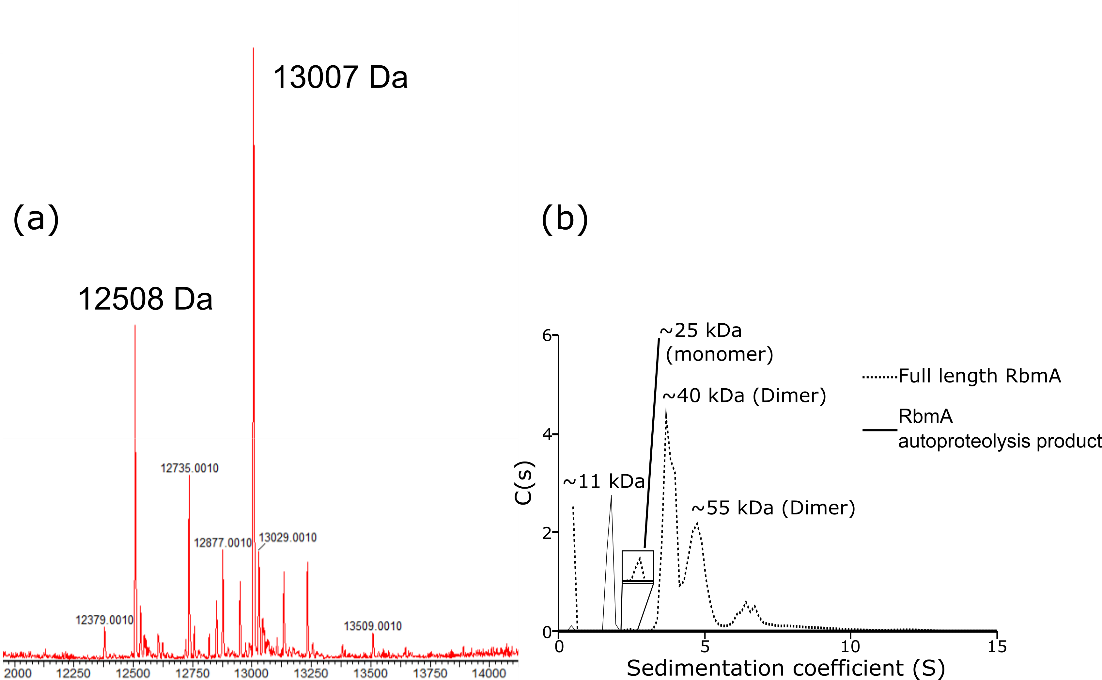
**

**Figure S1**. **Characterization of the RbmA fragmentation product.** (a) Mass spectrum of the low molecular weight RbmA proteolysis species showing the two main peaks at 12.5 and 13.0 kDa. (b) AUC analysis of the low molecular weight RbmA proteolysis species (solid line) compared to full length RbmA (dotted line). Both of these were prepared in the absence of MgPO_4_. The full length RbmA distribution is the same as shown on Fig. 2c.

**
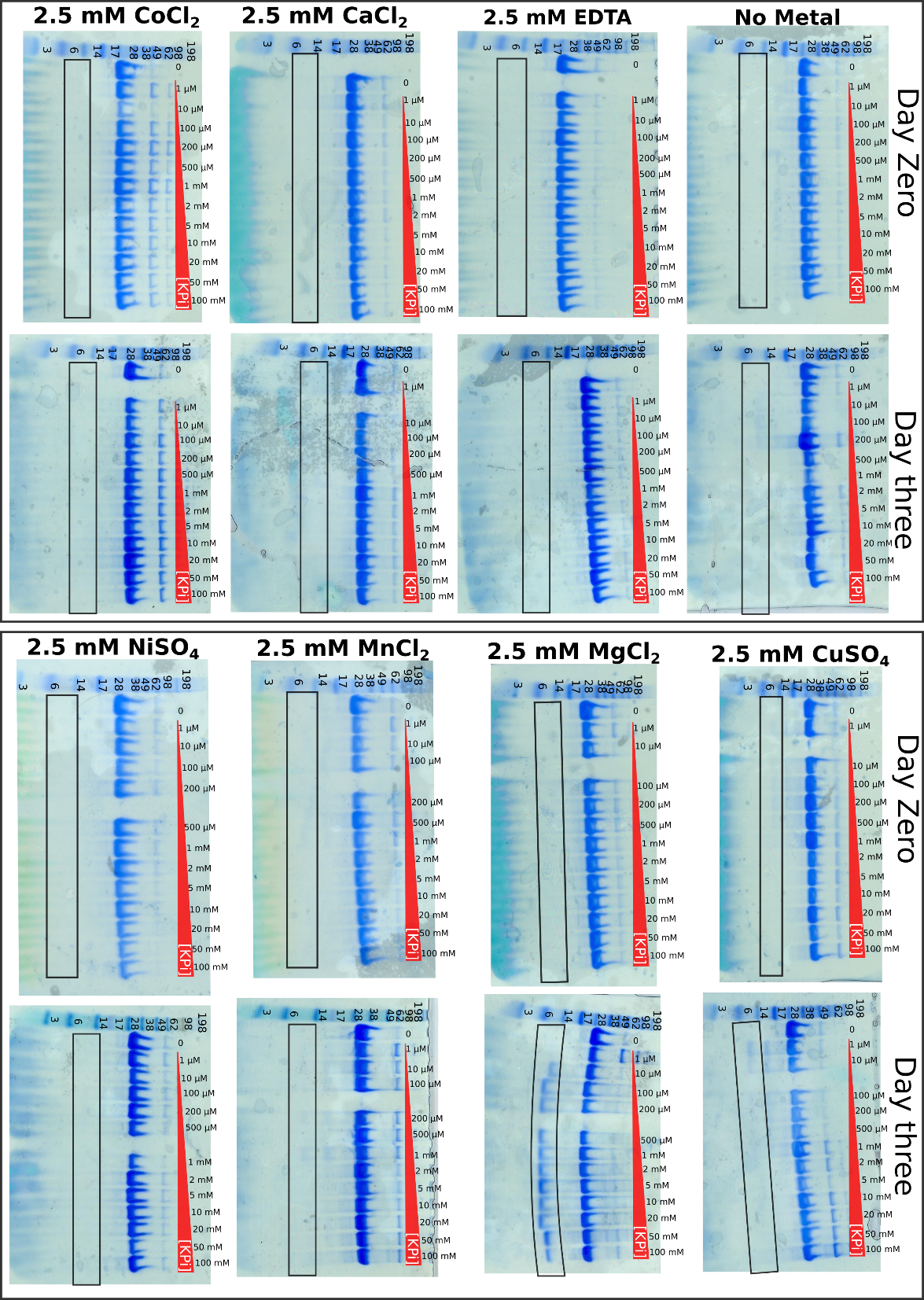
**

**Figure S2. Exploring RbmA proteolysis dependency on phosphate concentration and presence of di-valent cations.** Phosphate concentrations (red wedge) were screened against different divalent cations at a constant 2.5 mM concentration. Samples were taken directly after preparing the samples (Day Zero) and after 72 hours (Day three), and loaded in SDS-gels. Samples were screened for the presence of the fibronectin III B-Domain in the region between 6 and 14 kDa (black rectangle). As a control for trace amounts of divalent cations in the reaction buffer, the assay was also performed in the presence of 2.5 mM EDTA.


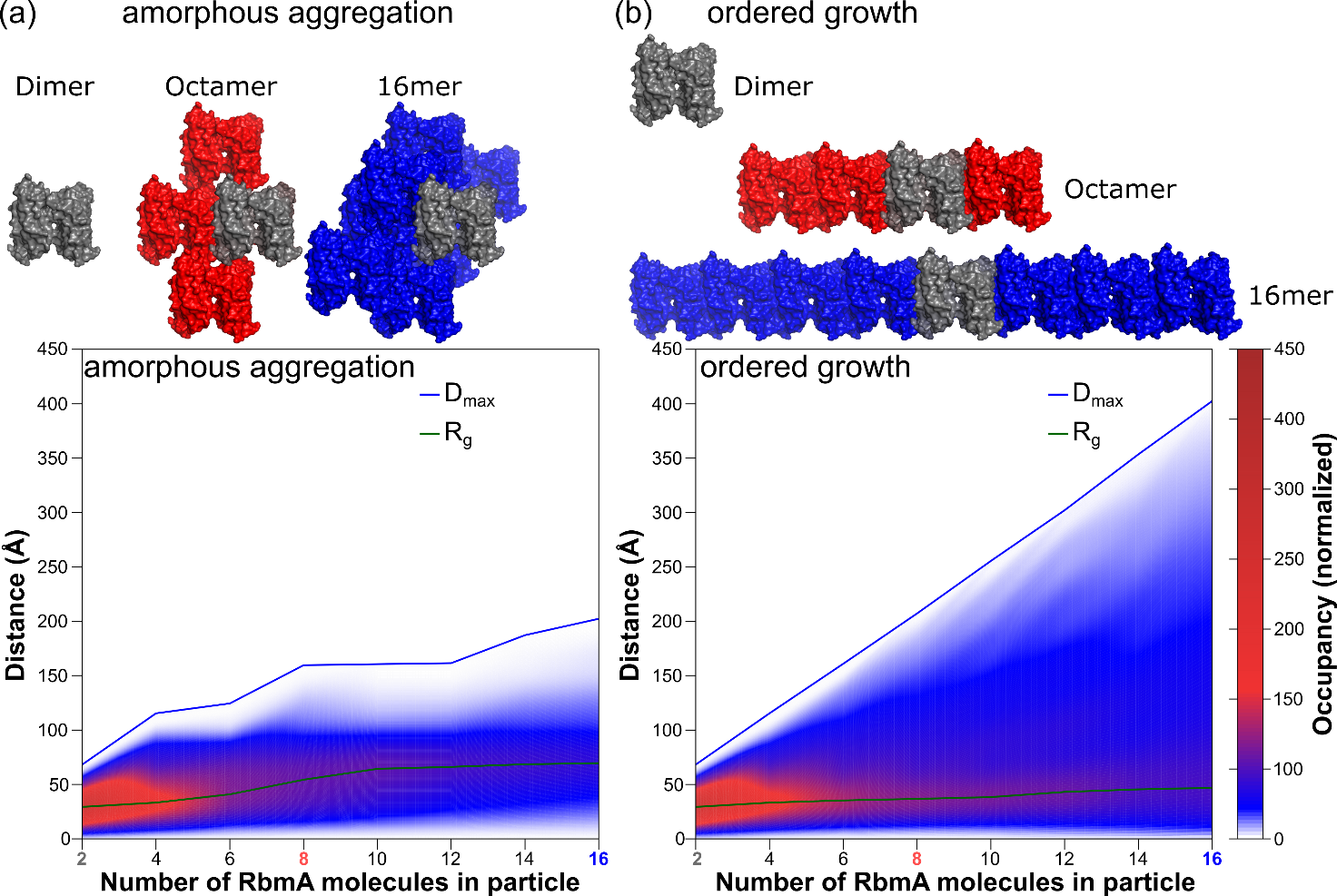


**Figure S3. Modeling amorphous aggregation versus ordered growth SAXS data.** (a) Model for amorphous aggregation. To model random aggregation, the RbmA crystallographic dimer (grey) was extended by positioning a copy of it at random on one of its faces. By repeating this process several times, it was possible to produce a randomly growing particle composed of an RbmA tetramer, hexamer, octamer (show in red, with the original dimer in grey), decamer, dodecamer, 14mer, and 16mer (shown in blue, with the original dimer in grey). A SAXS scattering profile was then calculated, and from it a pair distribution function. These were then plotted as a heat map, analogously to those in the main text. Further, the calculated radius of gyration (R_g_) and maximum distance (D_max_) were plotted as a green and blue line, respectively. (b) Model for ordered growth. To generate RbmA multimer particles which grew in an orderly fashion, the RbmA crystallographic dimer (grey) was extended by positioning a copy of it on an exposed tight-groove face. Accordingly, the particle could only extend in one direction, as shown exemplarily here for the octamer (red with the original grey dimer) and the 16mer (blue with the original grey dimer). *In silico* SAXS data was then generated and plotted as in (a). To note here is that in (a) both D_max_ and R_g_ change significantly during the process of random aggregation, while in (b) D_max_ changes dramatically, while R_g_’s change is rather small.

**
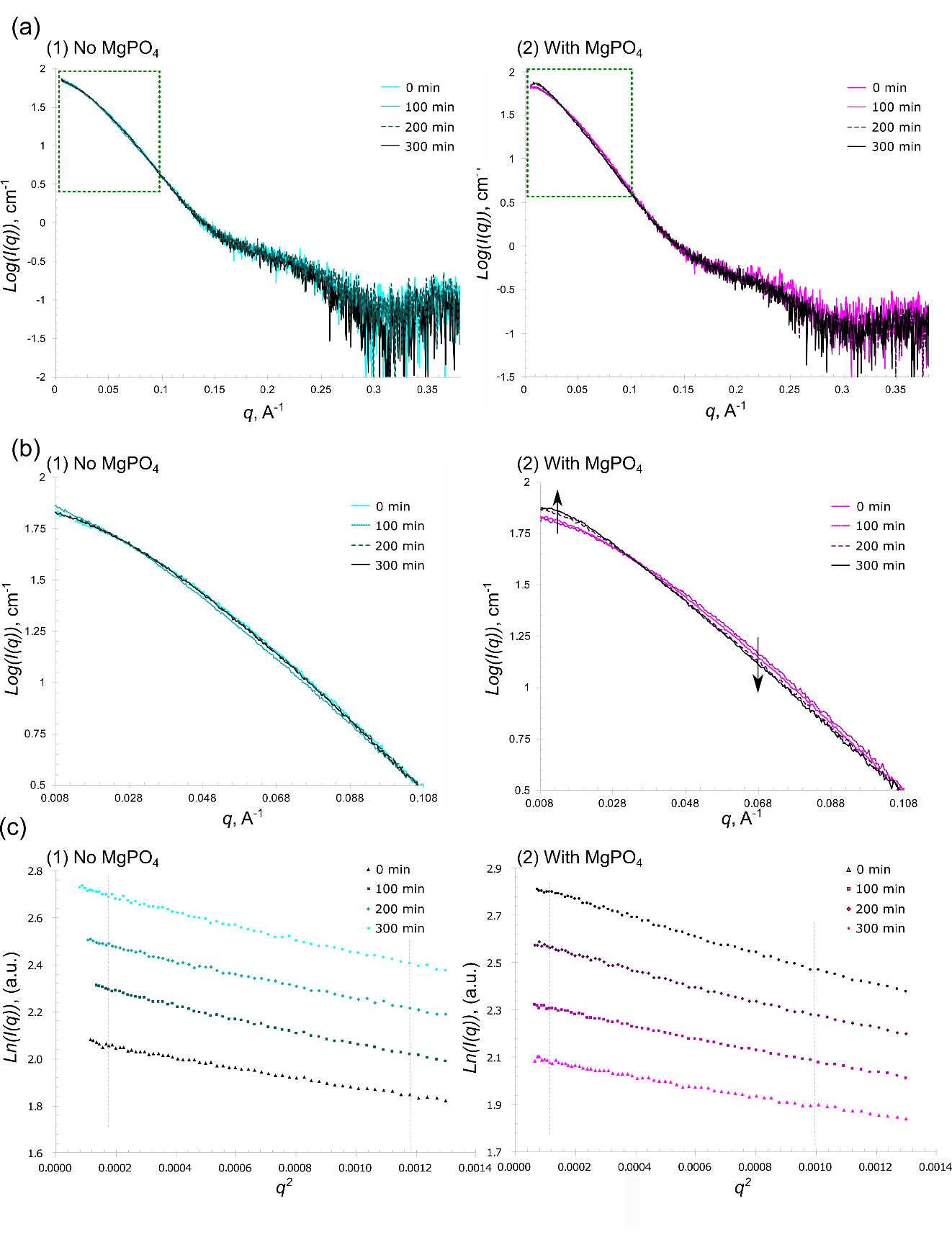
**

**Figure S4. Analysis of TR-SAXS data for RbmA wild type.** (a) Small-angle scattering curves of RbmA in the absence (left) and presence (right) of magnesium and phosphate ions. Green boxes are zoomed in and represented in (b). (c) Guinier analysis of time-, and MgPO_4_-dependent SAXS data of RbmA.

**
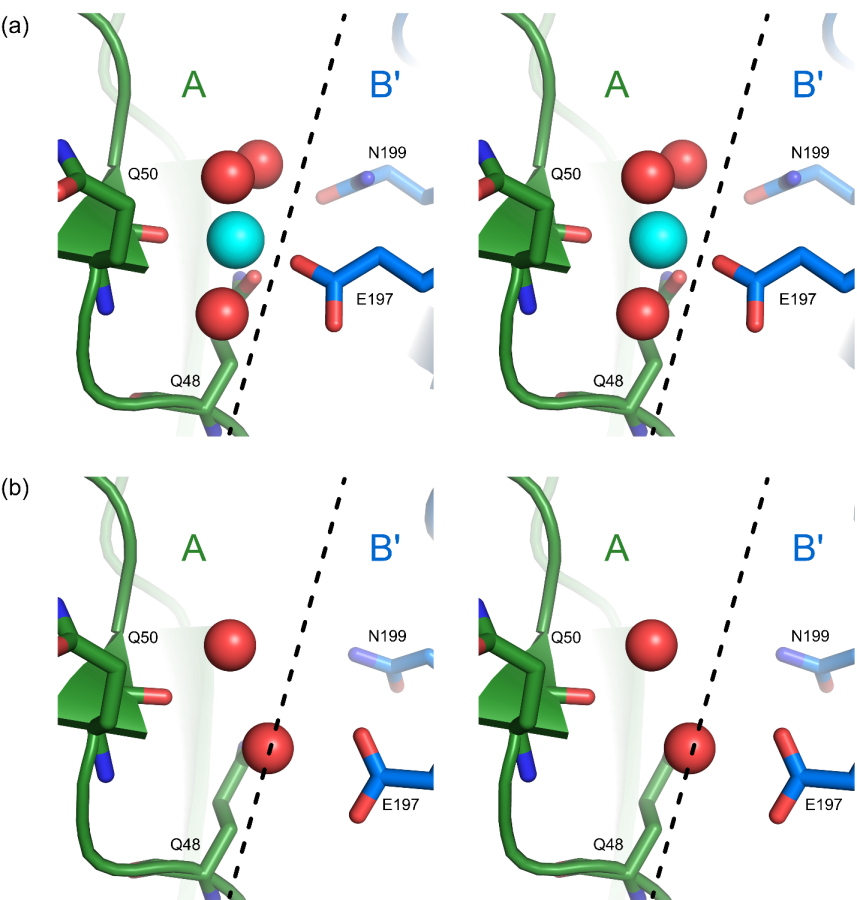
**

**Figure S5.** Stereo view of magnesium dependent crystal interface in RbmA crystals. In both figures, the chain A of the asymmetric unit in the unit cell origin is shown in green (indicated as A in both panels), while the B-chain of the symmetry mate in blue (indicated as B'); symmetry axis is highlighted as a dashed line. (a) Crystals grown in the presence of 10 mM magnesium sulfate show an octahedral magnesium cluster embedded between symmetry mates. Magnesium is shown as a cyan sphere, while water molecules as red spheres. (b) In crystals grown without magnesium sulfate, the area presents much weaker crystal contacts.


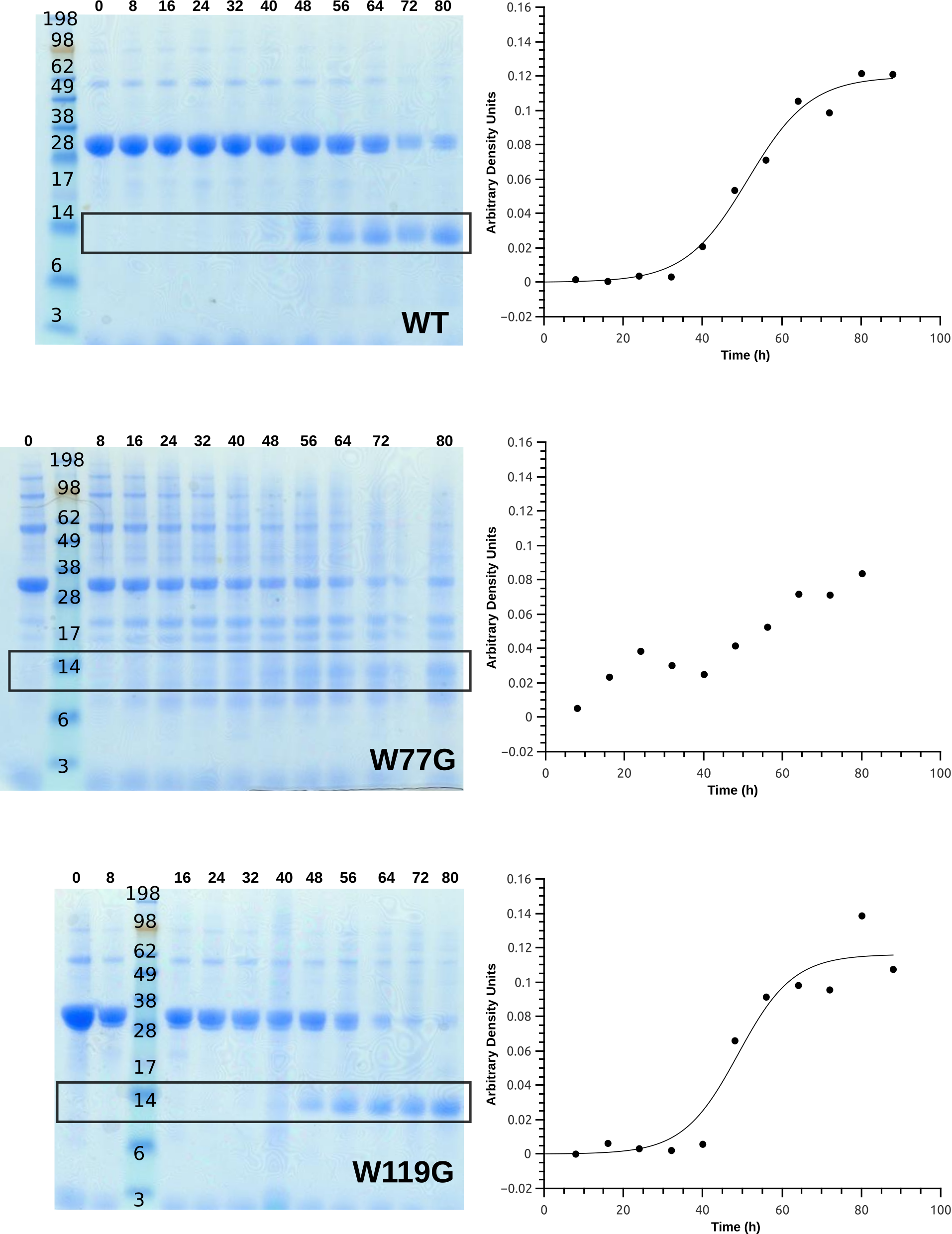


**Figure S6a.** RbmA magnesium phosphate dependent auto-proteolysis on various mutants scanning. Each gel sample corresponds to 5 μg total protein at time 0, with data taken every 8 hours for 80 hours (left). The area at which the low molecular band appeared is marked here with a black square. Appearance of the low molecular weight band was quantified via gel densitometry, plotted, and fitted following an autocatalytic mechanism (right). Each mutant is described at the bottom right of the corresponding SDS-PAGE. Samples that degraded too slowly, or not at all, were not fitted.


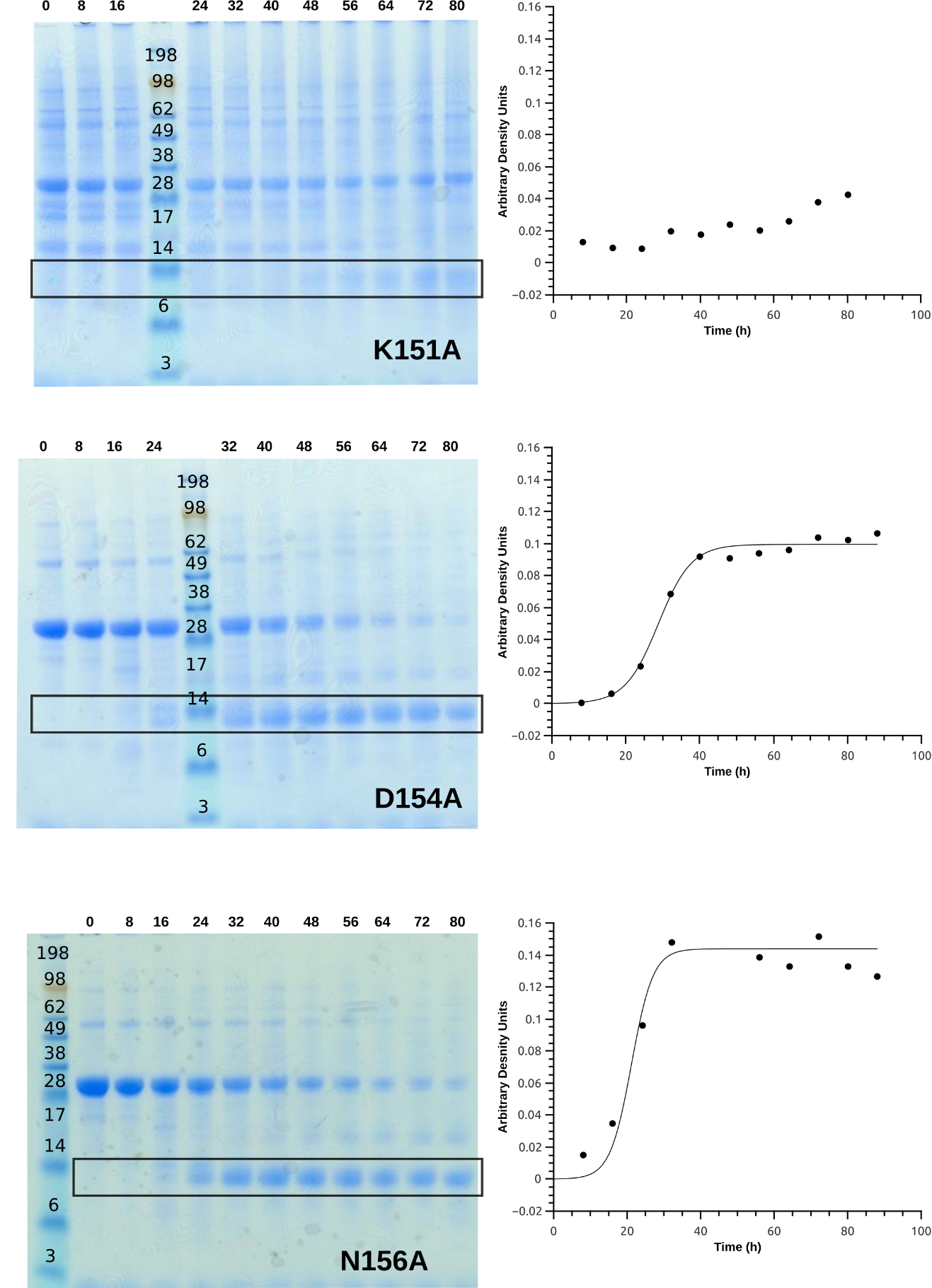


**Figure S6b.** RbmA magnesium phosphate dependent auto-proteolysis on various mutants scanning. Each gel sample corresponds to 5 μg total protein at time 0, with data taken every 8 hours for 80 hours (left). The area at which the low molecular band appeared is marked here with a black square. Appearance of the low molecular weight band was quantified via gel densitometry, plotted, and fitted following an autocatalytic mechanism (right). Each mutant is described at the bottom right of the corresponding SDS-PAGE. Samples that degraded too slowly, or not at all, were not fitted.


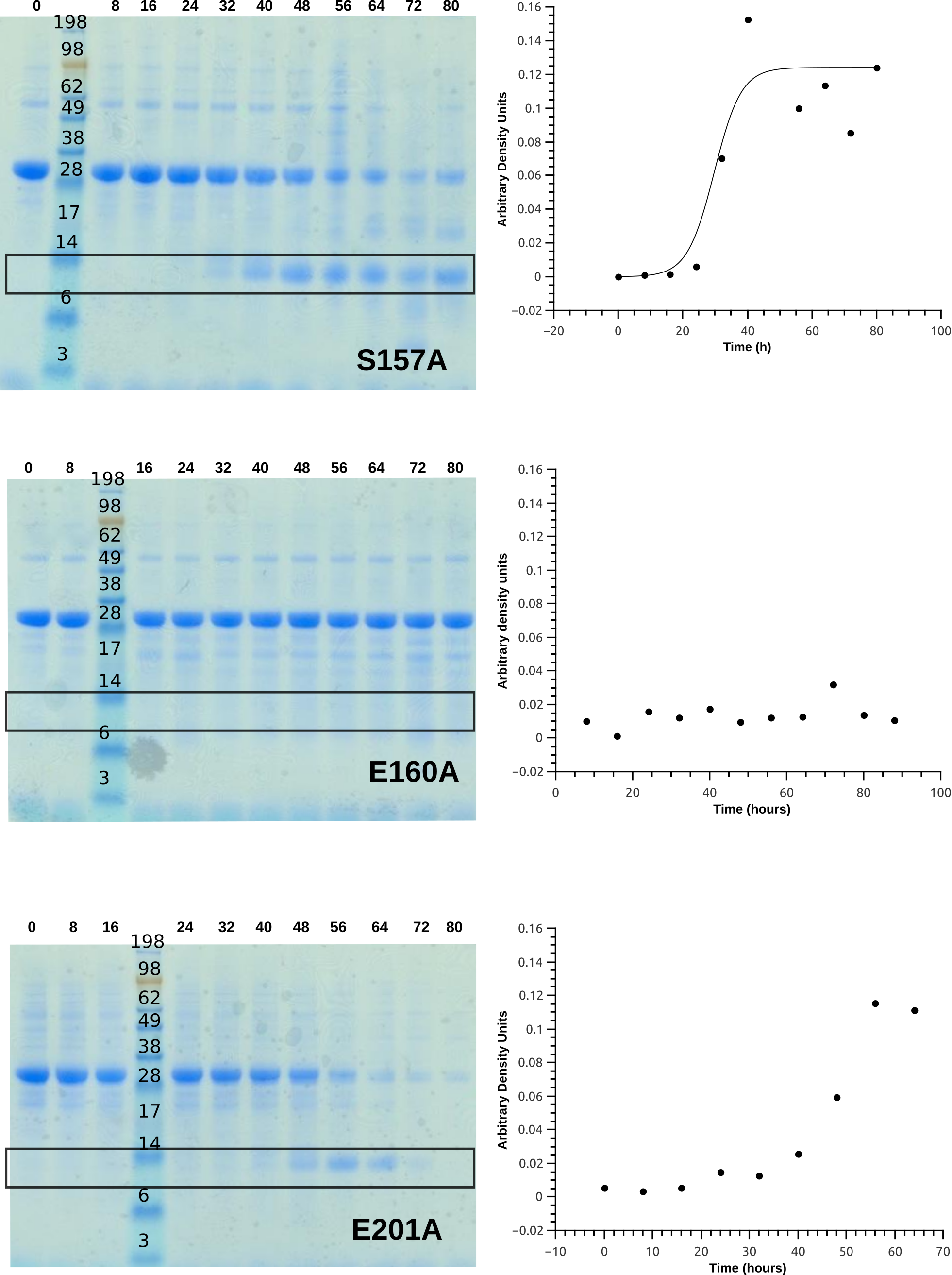


**Figure S6c.** RbmA magnesium phosphate dependent auto-proteolysis on various mutants scanning. Each gel sample corresponds to 5 μg total protein at time 0, with data taken every 8 hours for 80 hours (left). The area at which the low molecular band appeared is marked here with a black square. Appearance of the low molecular weight band was quantified via gel densitometry, plotted, and fitted following an autocatalytic mechanism (right). Each mutant is described at the bottom right of the corresponding SDS-PAGE. Samples that degraded too slowly, or not at all, were not fitted.


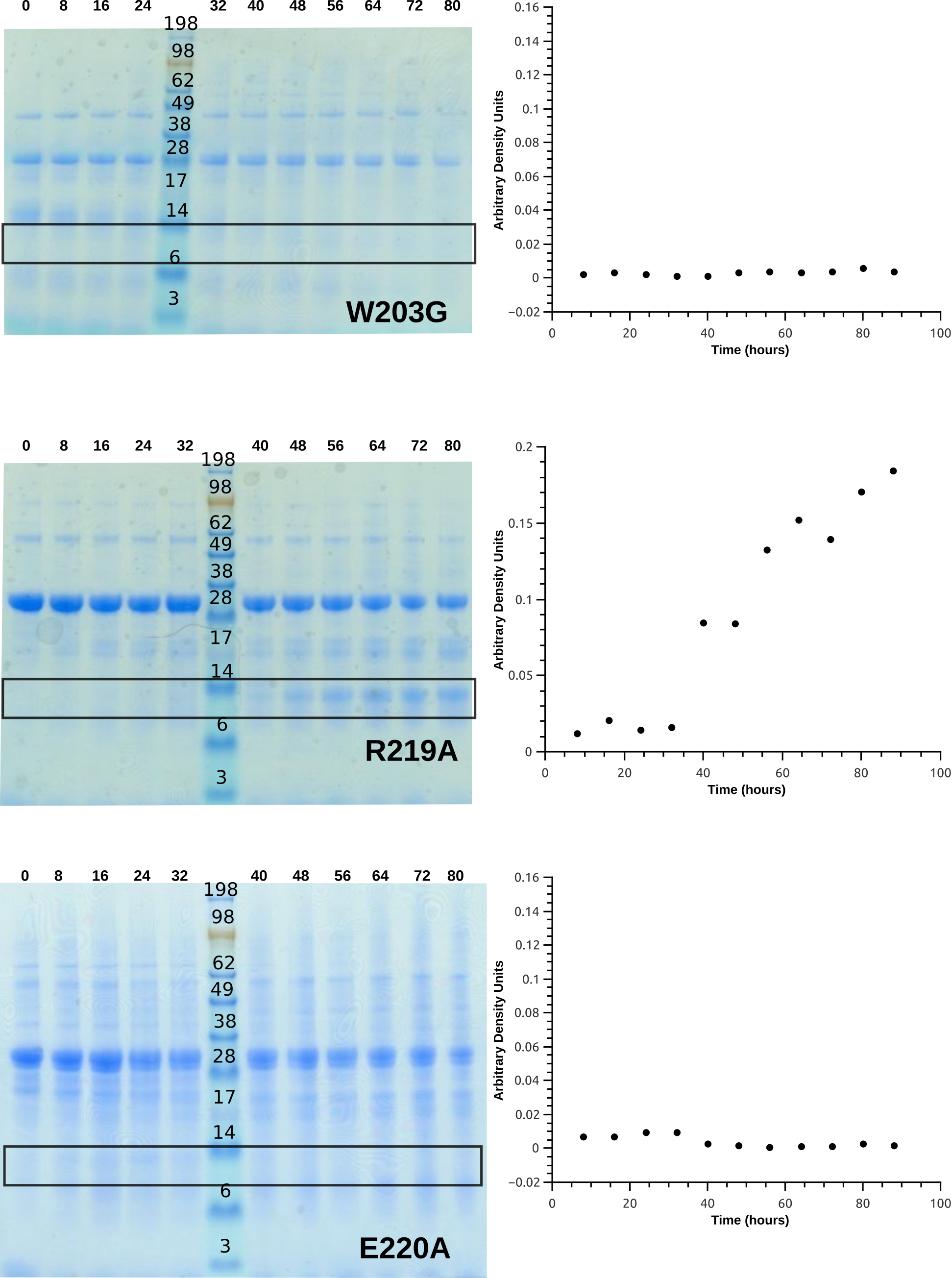


**Figure S6d.** RbmA magnesium phosphate dependent auto-proteolysis on various mutants scanning. Each gel sample corresponds to 5 μg total protein at time 0, with data taken every 8 hours for 80 hours (left). The area at which the low molecular band appeared is marked here with a black square. Appearance of the low molecular weight band was quantified via gel densitometry, plotted, and fitted following an autocatalytic mechanism (right). Each mutant is described at the bottom right of the corresponding SDS-PAGE. Samples that degraded too slowly, or not at all, were not fitted.


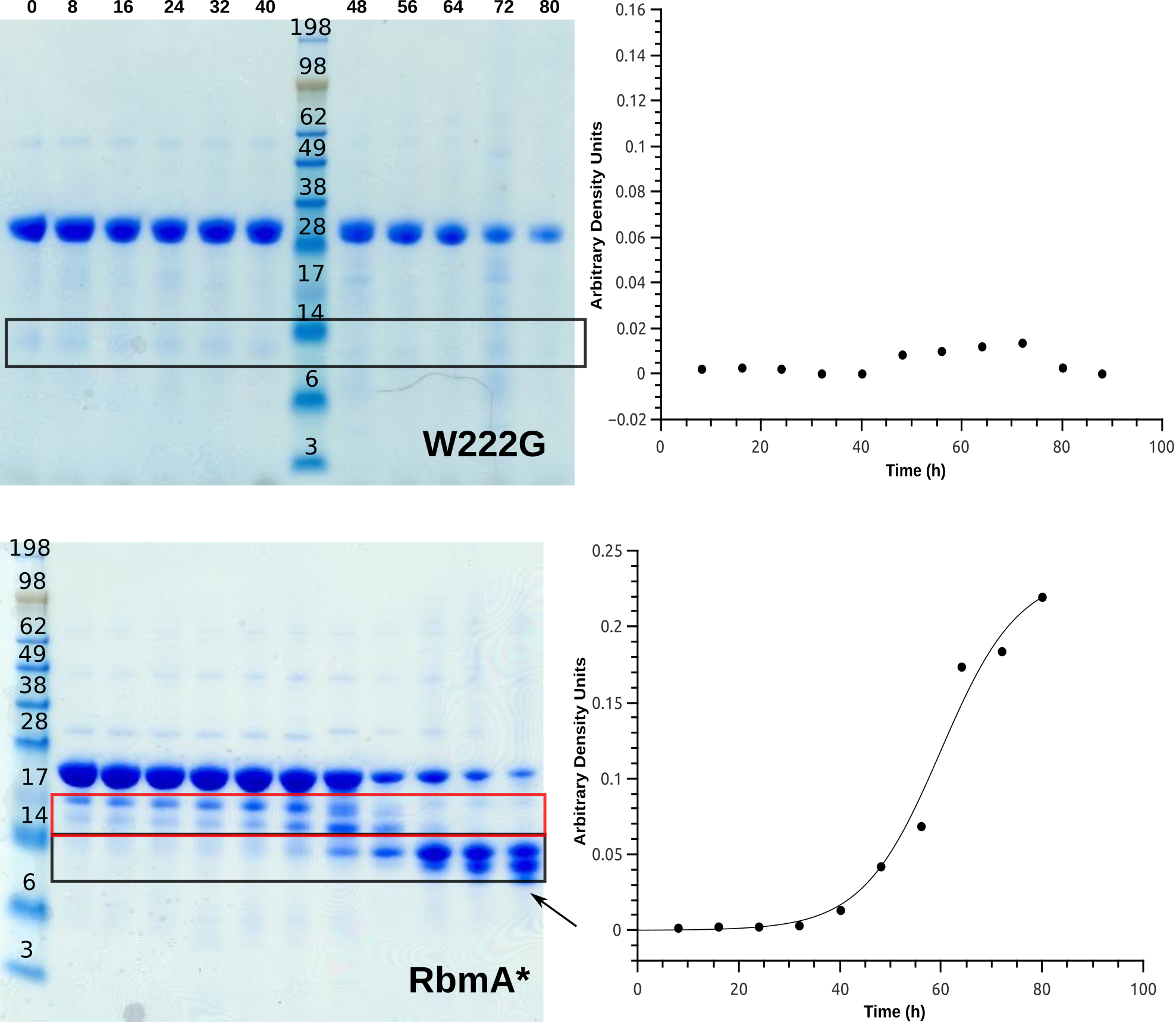


**Figure S6e.** RbmA magnesium phosphate dependent auto-proteolysis on various mutants scanning. Each gel sample corresponds to 5 μg total protein at time 0, with data taken every 8 hours for 80 hours (left). The area at which the low molecular band appeared is marked here with a black square. Appearance of the low molecular weight band was quantified via gel densitometry, plotted, and fitted following an autocatalytic mechanism (right). Each mutant is described at the bottom right of the corresponding SDS-PAGE. Samples that degraded too slowly, or not at all, were not fitted.


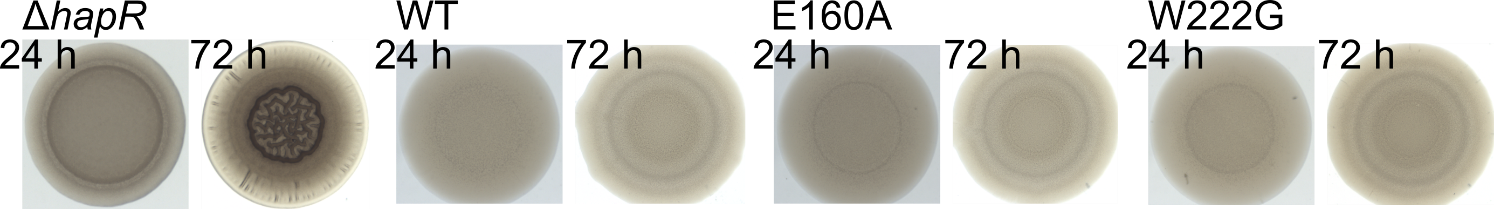


**Figure S7.** *V. cholerae* colonies. To monitor colony rugosity *V. cholerae* microcolony biofilms were grown as colonies on LB agar and imaged after 24 h and 72 h of growth. The Δ*hapR* strain is used as a positive control for the rugosity phenotype.

**
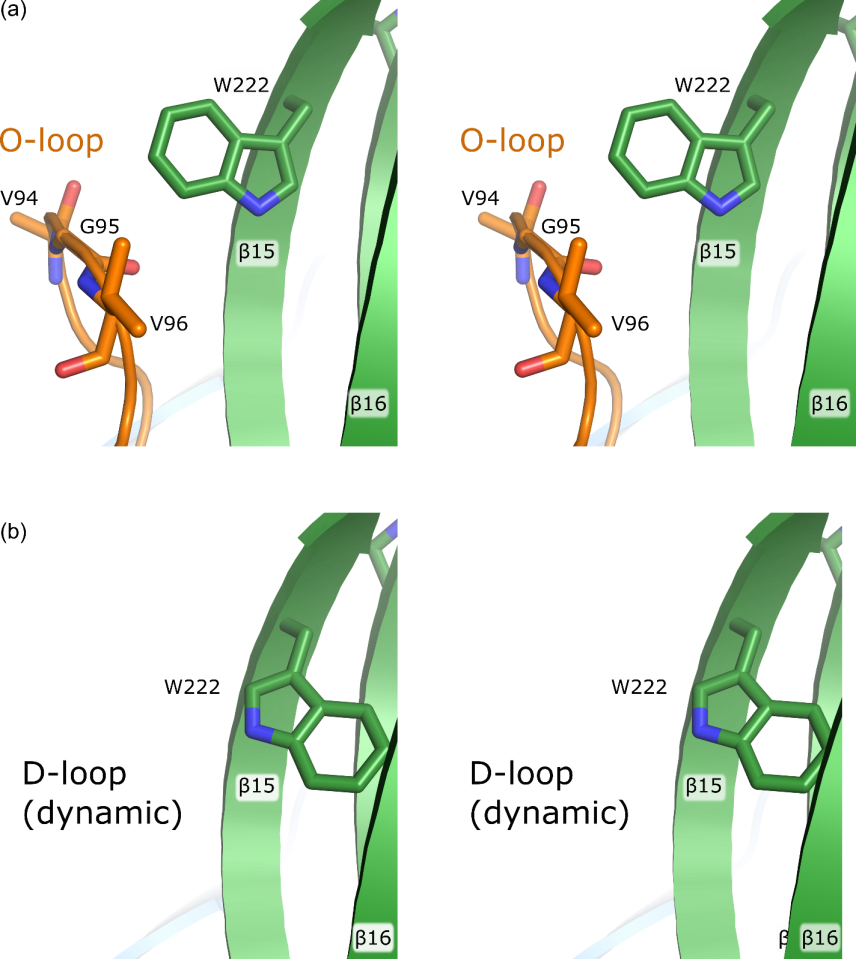
**

**Figure S8.** Stereo view of the conformational changes of W222. Detailed view of the FnIII-B domain β15 and β16 strands, with W222 shown as a stick figure. The O-loop conformation is highlighted in orange. (a) In the O-loop conformation, W222 flips out of the β-sandwich, and interacts with a hydrophobic patch on the O-loop (V94-G95-V96). (b) In the D-loop conformation, W222 is buried between the β15 and β16 strands.


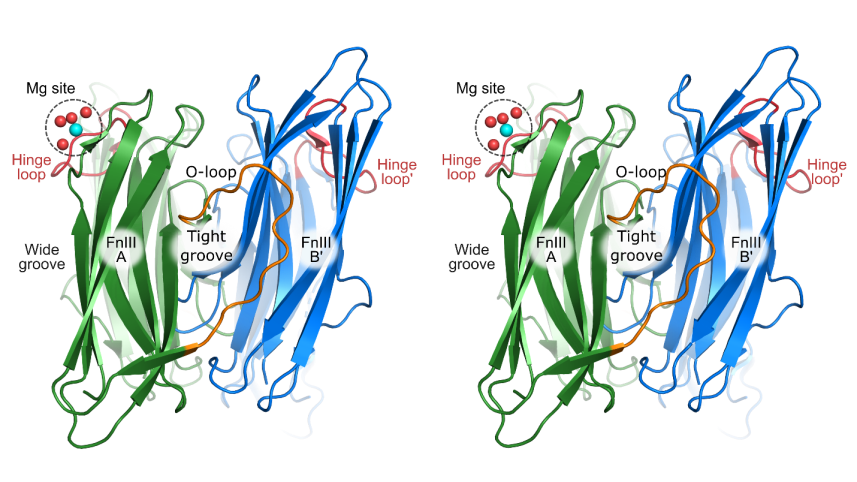


**Figure S9.** Stereo view of the overall topology of RbmA, as seen in Fig. 3a.

**Table S1.** Summary of protein parameters shown in figures 1, 2, 3 and 5.

|  | Kinetics | Thermofluor | SEC-SAXS (Å) | | | | | | | |
| --- | --- | --- | --- | --- | --- | --- | --- | --- | --- | --- |
| Protein | T1/2 (h) | Melting Temperature (˚C) | Peak I | | Peak II | | Peak III | | Peak IV | |
|  |  |  | R_g_ | D_max_ | R_g_ | D_max_ | R_g_ | D_max_ | R_g_ | D_max_ |
| WT | 51.3±2.9 | 48.5±0.0 | 32.9±1.4 | 116.6±8.3 | none | none | none | none | none | none |
| W77G | >80 | linear | 96.0±11.4 | 318.8±44.5 | 52.8±6.0 | 177.8±23.4 | none | none | none | none |
| W119G | 48.9±5.4 | 48.0±0.5 | 29.5±0.6 | 89.8±4.0 | 26.2±1.8 | 80.8±5.6 | none | none | none | none |
| K151A | >80 | linear | 63.4±6.3 | 195.5±21.3 | none | none | none | none | none | none |
| D154A | 28.8±1 | 44.5±0.0 | 30.4±0.6 | 96.2±11.9 | none | none | none | none | none | none |
| N156A | 21.2±1.6 | 46.5±0.0 | 31.6±0.9 | 104.1±7.9 | none | none | none | none | none | none |
| S157A | 29.8±5.8 | 46.3±0.3 | 30.8±0.8 | 101.1±5.8 | none | none | none | none | none | none |
| E160A | Inactive | 48.0±0.0 | 28.4±0.4 | 88.4±5.5 | none | none | none | none | none | none |
| E201A | 48.3±7.1 | 46.0±0.0 | 33.0±1.1 | 110.9±7.4 | none | none | none | none | none | none |
| W203G | Inactive | 45.0±0.5 | 31.6±1.5 | 104.6±8.7 | none | none | none | none | none | none |
| R219A | >80 | 44.5±0.0 | 33.5±1.9 | 105.9±5.9 | 25.8±1.5 | 83.9±7.0 | none | none | none | none |
| E220A | Inactive | 39±0.0 | 30.0±1.1 | 93.4±6.7 | none | none | none | none | none | none |
| W222G | Inactive | 46.3±0 | 31.1±0.7 | 102.4±6.4 | none | none | none | none | none | none |
| RbmA* | 59.5±3.1 | 44.5±2.2 | 52.2±3.0 | 155.1±7.6 | 46.8±0.4 | 144.2±6.4 | 41.7±1.9 | 138.2±7.2 | 43.6±3.0 | 152.8±15.8 |

**Table S2**. Primers employed to generate mutants.

| Mutant |  | Primer |
| --- | --- | --- |
| W77G | Forward | GTGAAATTTAAGTATGGGCTGAGCATTAAAGGCC |
|  | Reverse | GGCCTTTAATGCTCAGCCCATACTTAAATTTCAC |
| K151A | Forward | CAATCAAGAATTCGTTGCAGGTGTCGATCTGAAC |
|  | Reverse | GTTCAGATCGACACCTGCAACGAATTCTTGATTG |
| D154A | Forward | GAATTCGTTAAAGGTGTCGCTCTGAACAGCCTGCCG |
|  | Reverse | CGGCAGGCTGTTCAGAGCGACACCTTTAACGAATTC |
| N156A | Forward | CGTTAAAGGTGTCGATCTGGCCAGCCTGCCGGAACTG |
|  | reverse | CAGTTCCGGCAGGCTGGCCAGATCGACACCTTTAACG |
| S157A | Forward | GGTGTCGATCTGAACGCCCTGCCGGAACTGAATGG |
|  | Reverse | CCATTCAGTTCCGGCAGGGCGTTCAGATCGACACC |
| P159A | Forward | CGATCTGAACAGCCTGGCGGAACTGAATGGCCTG |
|  | Reverse | CAGGCCATTCAGTTCCGCCAGGCTGTTCAGATCG |
| E160A | Forward | CTGAACAGCCTGCCGGCACTGAATGGCCTGACG |
|  | Reverse | CGTCAGGCCATTCAGTGCCGGCAGGCTGTTCAG |
| E201A | Forward | GGTGAAGCAAACGTTGCATTCTGGATGACCG |
|  | Reverse | CGGTCATCCAGAATGCAACGTTTGCTTCACC |
| W203G | Forward | GAAGGTGAAGCAAACGTTGAATTCGGGATGACCGCAGTCGGTCCGGACGG |
|  | Reverse | CCGTCCGGACCGACTGCGGTCATCCCGAATTCAACGTTTGCTTCACCTTC |
| R219A | Forward | CCCGGTGAATGCGGCTGAAAAATGGGTTATTGC |
|  | Reverse | GCAATAACCCATTTTTCAGCCGCATTCACCGGG |
| E220A | Forward | GTGAATGCGCGTGCAAAATGGGTTATTGCC |
|  | Reverse | GGCAATAACCCATTTTGCACGCGCATTCAC |
| W222G | Forward | CGGTGAATGCGCGTGAAAAAGGGGTTATTGCCAGTGGCGATAC |
|  | Reverse | GTATCGCCACTGGCAATAACCCCTTTTTCACGCGCATTCACCG |
| FnIII-B-domain | Forward | GGTGGACATATGAACAGCCTGCCGGAAC |
|  | Reverse | CGCCGCCTCGAGTCACTTCTTCAC |

**Table S3.** Statistics for the X-ray structure of the RbmA-Mg^2+^ complex. Details for highest resolution shell in parenthesis.

|  | RbmA-Mg |
| --- | --- |
| PDB ID | 5G50 |
| **Data collection** | |
| Wavelength (Å) | 1.00 |
| Space group | *P4_1_2_1_2* |
| Unit Cell | *a*=*b =* 123.513 Å, *c* = 100.526, *α=β=γ= 90* º |
| Resolution range (Å) | 46.56 - 2.302 (2.384 - 2.302) |
| R_merge_ | 0.03104 (0.4064) |
| *I*/σ*I* | 17.16 (1.90) |
| Total reflections | 69082 (6583) |
| Unique reflections | 34970 (3394) |
| Completeness (%) | 100 (98) |
| Redundancy | 2.0 (1.9) |
| Wilson B-factor | 43.75 |
| **Refinement** | |
| Reflections used in refinement | 34911 (3394) |
| Reflections used for R-free | 1690 (166) |
| R_work_/R_free_ | 0.2055/0.2353 |
| No.of atoms | 3795 |
| Protein | 3464 |
| Ligands | 1 |
| Water | 330 |
| B-factors |  |
| Protein | 47.70 |
| Ligand | 48.18 |
| Water | 46.40 |
| Protein residues | 462 |
| R.M.S. deviations |  |
| Bond lengths (Å) | 0.012 |
| Bond angles (º) | 1.47 |

**Table S4.** Oligonucleotides used for *V. cholerae* strain construction

| **Oligo name** | **Sequence*** | **Description** |
| --- | --- | --- |
| KDO1281 | CAAACCATTGAGcgCAGGTAGGCTATTTAAATCGACTCC | For *rbmA^E160A^* mutation |
| KDO1282 | CCTGcgCTCAATGGTTTGACTATTGATATCAAAAATCAATTTGG | For *rbmA^E160A^* mutation |
| KDO1283 | GTATCACCTGAAGCAATAACtccTTTCTCTCTCGCATTGACAGG | For *rbmA^W222G^* mutation |
| KDO1284 | GAAAggaGTTATTGCTTCAGGTGATACTTATTCAAAAGTGCG | For *rbmA^W222G^* mutation |
| KDO865 | GTCAGGTGCAAAACTTCTGTT | For sequencing |
| KDO866 | CCTCAGTTTTGAAGGACTGAATAA | For sequencing |
| KDO280 | GACAACAAGCCAGGGATGTAACGCA | For sequencing and cloning |
| KDO281 | GGTGCTTAACCGTAAGTCTGACGA | For sequencing and cloning |

*mutant regions are given in lower case
